# Non-Skewed X-inactivation Results in NF-κB Essential Modulator (NEMO) Δ-exon 5-autoinflammatory Syndrome (NEMO-NDAS) in a Female with Incontinentia Pigmenti

**DOI:** 10.1007/s10875-024-01799-2

**Published:** 2024-09-12

**Authors:** Jessica Eigemann, Ales Janda, Catharina Schuetz, Min Ae Lee-Kirsch, Ansgar Schulz, Manfred Hoenig, Ingrid Furlan, Eva-Maria Jacobsen, Julia Zinngrebe, Sarah Peters, Cosima Drewes, Reiner Siebert, Eva-Maria Rump, Marita Führer, Myriam Lorenz, Ulrich Pannicke, Uwe Kölsch, Klaus-Michael Debatin, Horst von Bernuth, Klaus Schwarz, Kerstin Felgentreff

**Affiliations:** 1 Master’s Program of Molecular Medicine, Medical Faculty of Ulm University, Ulm, Germany; 2 Department of Pediatrics and Adolescent Medicine, Ulm University Medical Center, Ulm, Germany; 3 Department of Pediatrics, Medical Faculty Carl Gustav Carus, Technische Universität Dresden, Dresden, Germany; 4 German Center for Child and Adolescent Health (DZKJ), Partner Site Leipzig/Dresden, Dresden, Germany; 5 German Center for Child and Adolescent Health (DZKJ), Partner Site Ulm, Ulm, Germany; 6 Department of Clinical Chemistry, Ulm University Medical Center, Ulm, Germany; 7 Institute of Human Genetics, Ulm University and Ulm University Medical Center, Ulm, Germany; 8 Institute for Clinical Transfusion Medicine and Immunogenetics Ulm, German Red Cross Blood Service Baden-Wuerttemberg - Hessen, Ulm, Germany; 9 Institute for Transfusion Medicine, University of Ulm, Ulm, Germany; 10 Department of Immunology, Labor Berlin – Charité Vivantes GmbH, Berlin, Germany; 11 Department of Pediatric Respiratory Medicine, Immunology and Critical Care Medicine, Charité - Universitätsmedizin Berlin, Corporate Nember of Freie Universität Berlin, Humboldt- Universität zu Berlin, Berlin Institute of Health, Berlin, Germany; 12 Berlin Institute of Health at Charité - Universitätsmedizin Berlin, Berlin, Germany; 13 Charité - Universitätsmedizin Berlin, corporate member of Freie Universität Berlin, Humboldt- Universität zu Berlin, Berlin Institute of Health (BIH), Berlin-Brandenburg Center for Regenerative Therapies (BCRT), Berlin, Germany; 14 German Center for Child and Adolescent Health (DZKJ), partner site Berlin, Berlin, Germany

**Keywords:** NEMO, Incontinentia pigmenti, Autoinflammation, Immunodeficiency, Non-skewed X-inactivation

## Abstract

**Purpose:**

Genetic hypomorphic defects in X chromosomal *IKBKG* coding for the NF-κB essential modulator (NEMO) lead to ectodermal dysplasia and immunodeficiency in males and the skin disorder incontinentia pigmenti (IP) in females, respectively. NF-κB essential modulator (NEMO) Δ-exon 5-autoinflammatory syndrome (NEMO-NDAS) is a systemic autoinflammatory disease caused by alternative splicing and increased proportion of NEMO-Δex5. We investigated a female carrier presenting with IP and NEMO-NDAS due to non-skewed X-inactivation.

**Methods:**

*IKBKG* transcripts were quantified in peripheral blood mononuclear cells isolated from the patient, her mother, and healthy controls using RT-PCR and nanopore sequencing. Corresponding proteins were analyzed by western blotting and flow cytometry. Besides toll-like receptor (TLR) and tumor necrosis factor (TNF) signaling, the interferon signature, cytokine production and X-inactivation status were investigated.

**Results:**

IP and autoinflammation with recurrent fever, oral ulcers, hepatitis, and neutropenia, but no immunodeficiency was observed in a female patient. Besides moderately reduced NEMO signaling function, type I interferonopathy, and elevated IL-18 and CXCL10 were found. She and her mother both carried the heterozygous variant c.613 C > T p.(Gln205*) in exon 5 of *IKBKG* previously reported in NEMO-deficient patients. However, X-inactivation was skewed in the mother, but not in the patient. Alternative splicing led to increased ratios of NEMO-Dex5 over full-length protein in peripheral blood cell subsets causing autoinflammation. Clinical symptoms partially resolved under treatment with TNF inhibitors.

**Conclusion:**

Non-skewed X-inactivation can lead to NEMO-NDAS in females with IP carrying hypomorphic *IKBKG* variants due to alternative splicing and increased proportions of NEMO-∆ex5.

**Supplementary Information:**

The online version contains supplementary material available at 10.1007/s10875-024-01799-2.

## Introduction

The NF-kB essential modulator (NEMO), also referred to as inhibitor of kB kinase (IKK) gamma (IKKγ), is encoded by the X chromosomal *IKBKG* gene and plays an important regulatory role in NF-kB signaling by mediating cellular responses of toll-like receptors (TLRs), members of the interleukin-1 receptor (IL-1) receptor family and tumor necrosis factor (TNF) receptors [1–3]. Together with IKKα and IKKβ kinases NEMO/IKKγ participates in the IKK complex of the canonical NF-κB pathway to enable inducible phosphorylation of inhibitors of κB (IκB) proteins [4, 5]. IκB phosphorylation leads to its degradative ubiquitination thus releasing NF-κB proteins into the cytoplasm. The latter translocate into the nucleus to induce transcription of genes involved in modulation of inflammation, immune response, cell adhesion, cell survival and development [6]. In addition, NEMO regulates the non-canonical IKK-related tank-binding kinase (TBK) 1 and inducible IKK (iIKK), which are involved inTLR3 and RIG-I-like receptors signaling to induce type I interferons (IFNα and IFNβ) essential for the antiviral immune response [7, 8].

Whereas amorphic variants of *IKBKG* are lethal in males, hypomorphic NEMO deficiency results in X-linked hypohidrotic ectodermal dysplasia with immunodeficiency (EDA-ID) composed of hypogammaglobulinemia, susceptibility to infections, ectodermal dysplasia, and various degrees of defective T- and B-cell function [9–12]. Female carriers present with the skin disorder incontinentia pigmenti (IP) [13], but retained immune function. However, immunodeficiency [14–17], as well as inflammatory disorders such as Behcet’s disease [18–20], have been reported in cases of non-skewed lyonization.

Skipping of exon 5 due to alternative splicing results in NEMO-∆ex5 associated autoinflammatory syndrome (NDAS), which was predominantly observed in young children presenting with panniculitis and systemic inflammation [21–23]. In contrast to patients affected by NEMO hypomorphism, patients presenting with NDAS carried splice variants in *IKBKG*. Although hypogammaglobulinemia has been reported in some of the patients, none developed severe immunodeficiency.

We report on a female patient presenting with IP and NEMO-NDAS due to a point mutation in exon 5 of *IKBKG* and non-skewed X-inactivation in peripheral blood cells.

## Methods

### Collection of Human Samples

This study was approved by the institutional ethics review board of Ulm University (144/20) and both parents, the patient, the patient mother and three adult controls gave informed consent to participate in this study and to the publication of this article.

Blood and serum samples were drawn from the patient and her mother at several clinical visits over a period of 4 years and 9 months. Peripheral blood mononuclear cells (PBMCs) were isolated using Ficoll Paque Plus™ (GE Healthcare).

### Type I Interferon (IFN) Signature in Peripheral Blood Cells

Gene expression of IFN-related genes was determined in PBMCs by quantitative RT-PCR and used for calculation of IFN scores as described before [24].

### RT-PCR and Sequencing of *IKBKG* Transcripts

RNA was isolated from PBMCs or sorted cell subsets, respectively, using TRI Reagent™ (Sigma-Aldrich) and GlycoBlue™ (Thermo Fisher Scientific), and transcribed into cDNA (SuperScript IV Reverse Transcriptase, Thermo Fisher Scientific).

RT-PCR, Sanger and nanopore sequencing methods are described in the supplementary methods and Table S1 and S2.

### Cloning of Transcript Variants and in-vitro Protein Synthesis

The WT, Mut, Dex4, Dex5, Dex4-5, and Dex4-6 *IKBKG* transcript variants were cloned into the pcDNA6 vector under the T7 promoter to enable further in-vitro synthesis of corresponding proteins (Table S3).

### Western Blotting of NEMO Proteins

Recombinant rabbit anti-IKK gamma/NEMO (ab188569, Abcam) (1:10000), purified mouse anti-IKK gamma/NEMO (611306, BD) (1:1000), and rabbit anti-GAPDH (ab181602, Abcam) (1:40000) were used as primary antibodies. Goat anti-rabbit IgG (H + L)-HRP conjugate (1706515, BioRad) (1:3000), and goat anti-mouse IgG (H + L)-HRP conjugate (1706516, BioRad) (1:5000) were used for detection with the SuperSignal™ West Pico Chemiluminescent Substrate (Thermo Fisher Scientific).

### Intracellular NEMO Staining, flow Cytometry and cell Sorting

Up to 10^5^ PBMCs were permeabilized and stained as described in the supplementary section. Samples were acquired on a FACS Aria I (BD) and analyzed according to the gating strategy shown in Figure S2A. T, B, NK lymphocytes, and monocytes were sorted from PBMCs on a FACS Aria I following immunofluorescent surface staining (supplementary methods).

### NEMO Functional Studies

Functional studies were performed on whole blood cells as described before [12], and on PBMCs obtained from the patient, her mother and two controls, respectively, as described in the supplementary section.

### X-inactivation Studies

To investigate potential skewing of DNA methylation at the X-chromosomal *IKBKG* locus we performed nanopore sequencing with calling of genomic variants and DNA methylation as detailed in the supplementary methods.

### Immunoassays for Cytokine Detection

The Proteome Profiler Human Cytokine Array Kit (R&D Systems) was used to investigate expression levels of 36 cytokines in serum samples obtained from patient, mother, and controls 1 and 2. Preparation of membranes and optical densitometry were performed as described for western blotting.

Concentrations of IL-18 were measured in serum samples using the IL-18 SimpleStep ELISA Kit (Abcam) on a POLARstar Omega (BMG Labtech) ELISA Reader.

### Statistical Analysis

Where applicable, data were graphed and analyzed using Prism Vs9. Statistical significance was indicated in figures as * *p* ≤ 0,05, ** *p* ≤ 0,01, *** *p* ≤ 0,001, **** *p* ≤ 0,0001.

## Results

### Case Report

**Fig. 1:**
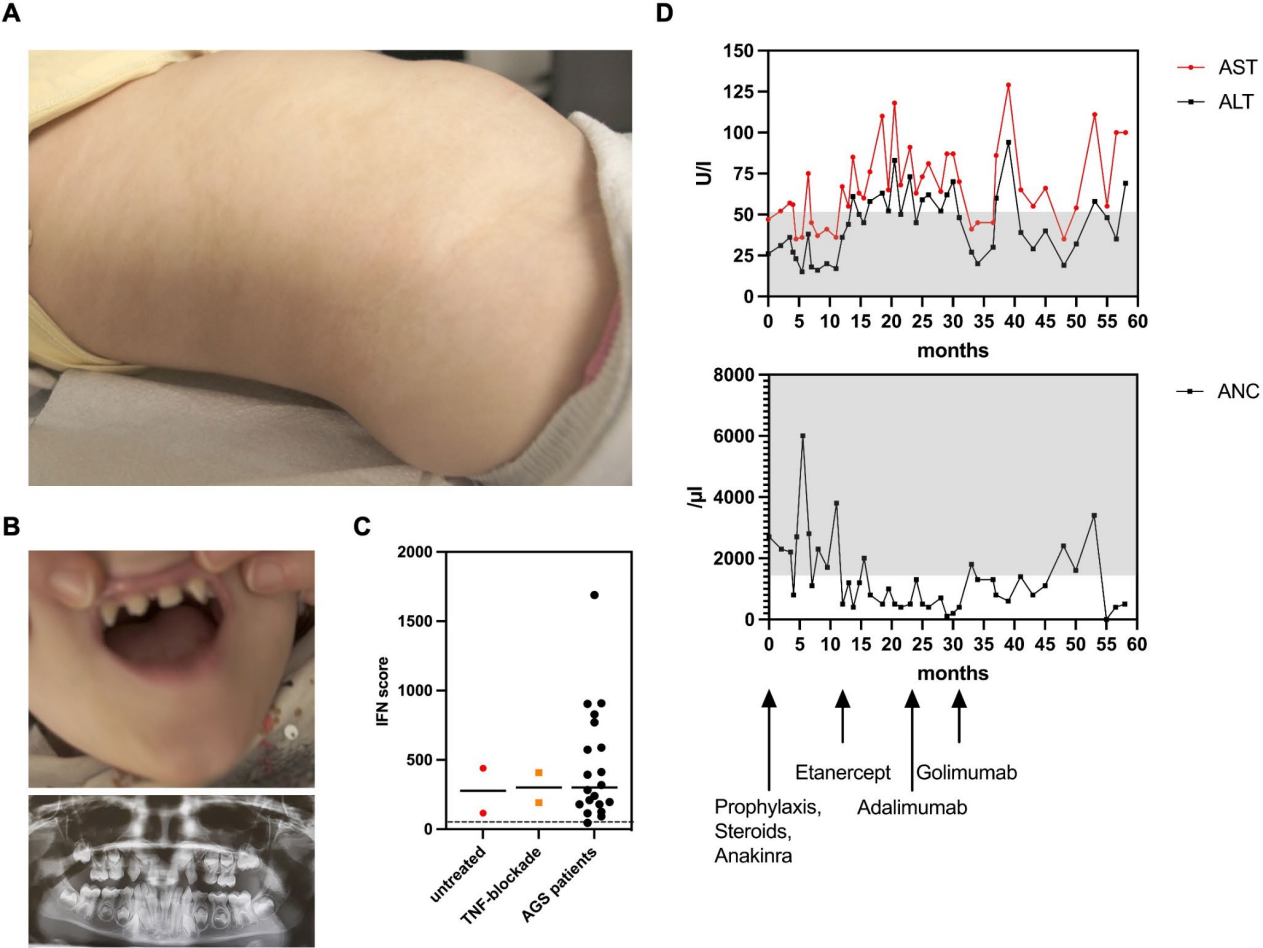
Clinical presentation of incontinentia pigmenti (IP) and NEMO-NDAS. At the age of 4y, the patient presented with ectodermal dysplasia characterized by hyperpigmentation along the Blashko lines (**A**) and conical teeth (**B**) (top: teeth at the age of 4y, bottom: dental X-ray at the age of 9y). Type I IFN-scores were calculated based on expression of IFN-stimulated genes analyzed in peripheral blood cells before start of treatment and under treatment with TNF inhibitors (**C**). IFN-scores of a cohort of 20 patients with typical interferonopathy (Aicardi-Goutières syndrome; AGS) are shown as control. Median values are displayed by horizontal bars. The cut-off for pathologic levels is indicated by the dotted line. Under treatment with TNF inhibitors as indicated, the patient developed mild hepatitis shown by elevated liver enzymes (ALT: alanine aminotransferase and AST: aspartate aminotransferase), and neutropenia (ANC: absolute neutrophil count) (**D**). Normal ranges are marked by grey-shaded areas

A female patient born as the first child to non-consanguineous parents of Turkish descent presented with recurrent fever episodes since the age of 12 months. Because the latter were associated with aphthous lesions and abdominal pain, she was clinically diagnosed with Familial Mediterranean Fever (FMF) despite no evidence of a disease-causing genetic variation in *MEFV*. However, the symptoms persisted under the treatment with colchicine. She was affected by IP, which was also present in her mother, and conical teeth (Fig. 1A, B). She had no history of severe infections and all vaccinations including BCG were well tolerated. Her family history was negative for infectious complications or autoinflammation.

When she was investigated in our center at the age of 4 years, molecular genetics could not confirm fever syndromes caused by variations in *MVK*,* NLRP3*, or *TNFRSF1A*. Inflammation parameters such as IL-1β, TNF and IL-2R were in the normal range. Serum amyloid A was elevated during a flare (129 mg/l; normal range < 10 mg/l), but in normal ranges at later visits.

Immunophenotyping including immunoglobulin levels, and lymphocyte subsets revealed mildly impaired B-cell maturation with reduced CD27^+^ memory B cell subsets (Table S4). Vaccination titers for tetanus, diphtheria, and pneumococcal disease were non-protective (Table S4), however, the vaccination status was incomplete. The type I IFN-regulated gene signature was increased to similar ranges as observed in a historical cohort of patients with Aicardi-Goutières syndrome (AGS), which persisted after start of treatment with TNF inhibitors (Fig. 1C). After the diagnosis of autoinflammatory syndrome NEMO-NDAS, the patient started treatment with etanercept, which was later changed to adalimumab. However, diffuse alopecia with eczema leading to recurrent bacterial super-infections occurred under the latter medication. Although fever episodes and oral ulcers stopped, the patient developed neutropenia and elevated liver enzymes over time (Fig. 1D). Autoantibodies targeting liver tissue or neutrophils were not detected. A treatment attempt with the JAK inhibitor tofacitinib was not successful due to reoccurring fever episodes.

### The p.Gln205* Variant Leads to Alternative Splicing and *IKBKG*-Δex5 Transcripts

Because of the clinical presentation with ectodermal dysplasia a genetic variation in the X-chromosomal *IKBKG* gene was assumed and the heterozygous variant c.613 C > T p.(Gln205*) (NM_003639) in exon 5 was found in the patient and her mother. This variant has been reported before in a male patient with NEMO immunodeficiency syndrome and his family members [20]. Further characterization of this variant revealed expression of NEMO-Δex5 due to alternative splicing.

**Fig. 2:**
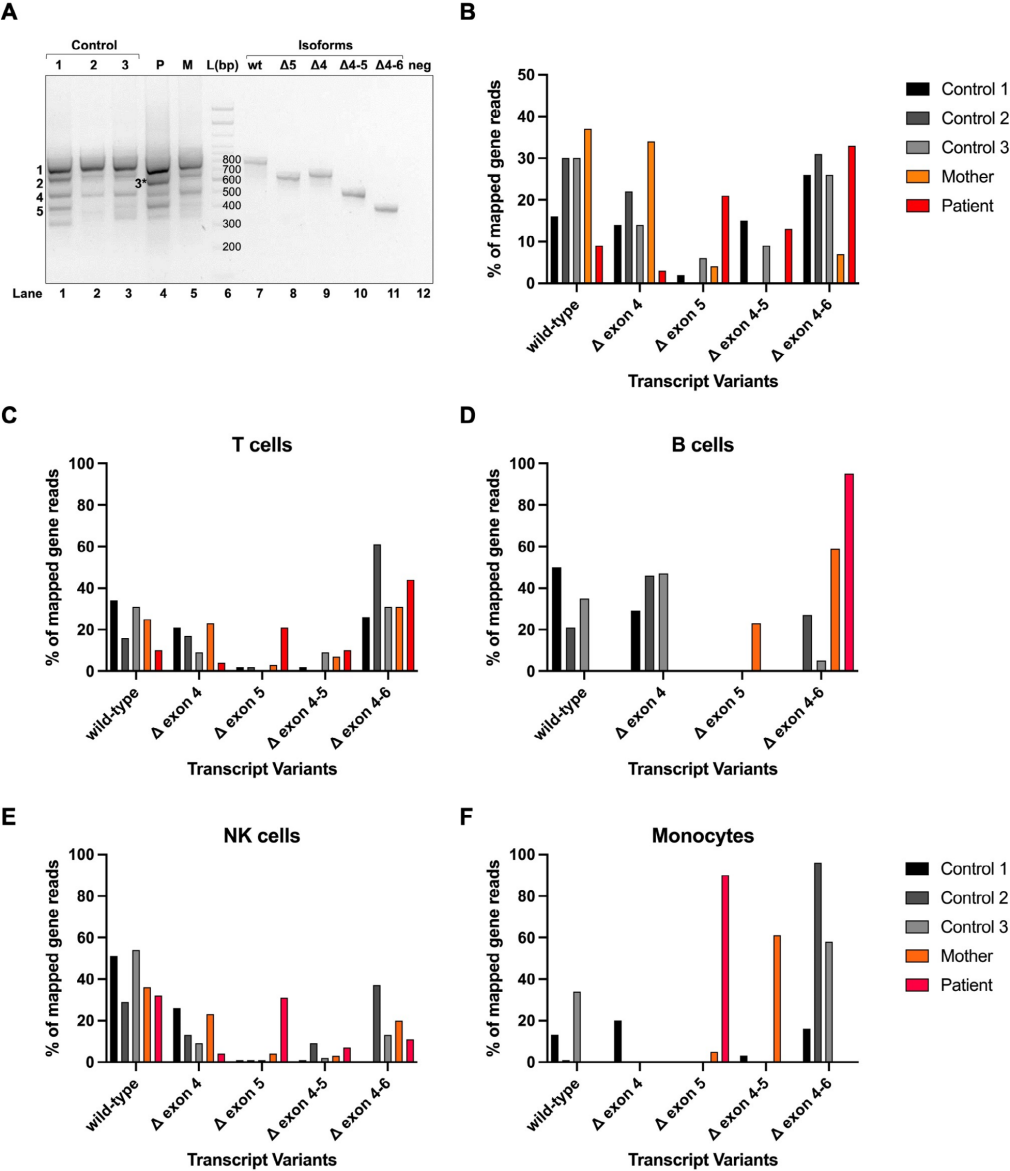
The variant c.613 C > T leads to expression of* IKBKG-*∆ex5 transcripts and NEMO-∆ex5 proteins. RNA was isolated from peripheral blood obtained from the patient, her mother and three healthy controls. *IKBKG* transcripts were amplified from cDNA and separated by electrophoresis (**A**). Full-length *IKBKG* transcripts (wt) and transcript variants ∆ex4 (∆4), ∆ex5 (∆5), ∆ex4-5 (∆4–5), ∆ex4-6 (∆4–6) were cloned into the pcDNA6 vector and used as size markers. In addition, bands obtained were excised and sequenced by Sanger sequencing: 1: wt (780 bp), 2: ∆4 (661 bp), 3 (*only observed in patient cells): ∆5 (627 bp), 4: ∆4–5 (508 bp), 5: ∆4–6 (411 bp). Transcript variants were further analyzed by nanopore sequencing and mapped gene reads were quantified using a library of all detected *IKBKG* transcripts. Shown are the frequencies of the most abundant variants ∆ex4, ∆ex5, ∆ex4-5, and ∆ex4-6 as % of all mapped gene reads obtained from whole PBMCs of patient, mother and controls (**B**). *IKBKG* transcripts were amplified from cDNA of T, B, NK lymphocytes, and monocytes and analyzed by nanopore sequencing (**C-F**). Shown are the frequencies of the most abundant variants Δex4, Δex5, Δex4-5, and Δex4-6 as % of all mapped gene reads. Of note, Δex4-5 could not be detected in B cells (**D**)

*IKBKG* transcripts were studied in peripheral blood cells obtained from the patient, her mother and 3 healthy controls. RNA was isolated from PBMCs, transcribed into cDNA and used for amplification of exons 3–8 (FigureS1A, B). Transcripts obtained by RT-PCR were separated by gel electrophoresis and identified by Sanger sequencing (Fig. 2A). Besides the full-length transcript variant 3 (NM_003639), several shorter transcripts were identified. The isoforms Δex4, Δex5, Δex4-5, and Δex4-6 were cloned into the pcDNA6 vector and used as size markers. Isoforms Dex4, and Dex4-5 result in disruption of the reading frame. Whereas the Δex4, Δex4-5, Δex4-6 isoforms could also be observed in the mother and the controls, Δex5 was predominantly found in patient cells.

In addition, *IKBKG* transcripts were quantified using Oxford Nanopore Sequencing. Besides the full-length transcript, Δex4, Δex5, Δex4-5, and Δex4-6 transcripts were found in considerable amounts in all individuals (Fig. 2B), although Δex5 and Δex4-6 variants were increasingly expressed in patient PBMCs. Variants observed with lower frequency are summarized in the transcript table (Table S2).

Frequencies of *IKBKG* transcripts were further studied in isolated T, B, and NK lymphocytes, and monocytes (Fig. 2C-F, Figure S1C). In T cells, mostly Δex5 and Δex4-6 transcripts were found in the patient, whereas Δex5 was much rarer in her mother and the controls. The frequencies of WT and Δex5 transcripts were almost similar in NK cells, whereas only Δex4-6, or Δex5 could be observed in patient B cells and monocytes, respectively. In total, fewer reads were obtained from B cells and monocytes due to lower cell counts resulting in lower RNA content obtained from these subsets.

In summary, the distribution of *IKBKG* transcripts was shifted in patient blood cells to a higher frequency of Δex5 transcripts at the expense of the full-length transcript.

### No Truncated Protein but NEMO-Δex5 is Expressed in Peripheral Blood Cells

Expression of NEMO proteins were studied in lysed PBMCs obtained from the patient, her mother and controls using western blot. Proteins of respective *IKBKG* transcripts were synthesized in vitro to be used as size marker (Table S5). Two antibodies with different target sites, abNEMO^150–300^ binding to exon 4–7 (amino acids 150–300), and abNEMO^278–396^ binding to exon 7–10, respectively, were used for detection (Figure S1B). Expression of synthesized proteins was confirmed using the two antibodies mentioned above and GAPDH as loading control (Figure S1D). Besides the full-length NEMO protein (48 kDa), the truncated protein synthesized from the c.613 C > T variant could be detected by abNEMO^150–300^, whereas abNEMO^278–396^ detected NEMO-Δex5 and NEMO-Δex4-6, but not the truncated protein (Figure S1 E). As mentioned above, Δex4 and Δex4-5 transcripts do not allow protein expression due to disruption of the reading frame.

**Fig. 3:**
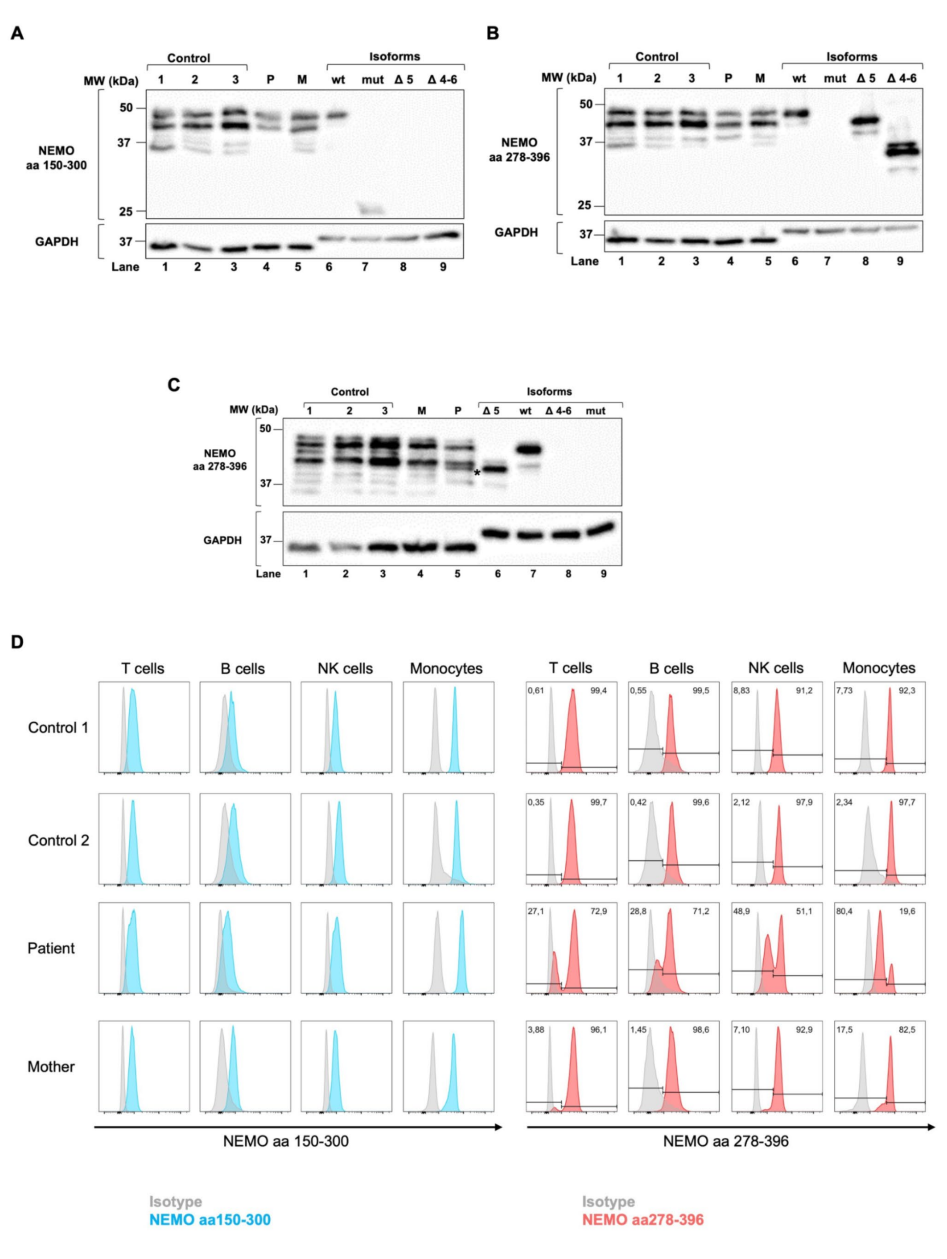
NEMO-∆ex5 is expressed in patient peripheral blood cells. Protein lysates obtained from PBMCs isolated from patient, mother and 3 controls were separated by SDS-PAGE and blotted on a PVDF membrane by western blotting. Proteins synthesized in-vitro from cloned full-length (wt), full-length with c.613 C > T variant in exon 5 (mut), ∆ex5 (∆5), and ∆ex4-6 (∆4–6) *IKBKG* transcripts were used as size markers in addition to a 10-250 kDa ladder. NEMO proteins were detected using primary antibodies binding to amino acids (aa) 150–300 (NEMO^150–300^) (**A**) and aa278-396 (NEMO^278–396^) (**B**,** C**).Besides the wt protein (48 kDa), several cross-reactional bands were detected by both antibodies, as described by the manufacturer. No protein could be detected from Δex4 and ∆ex4-5 transcripts due to disruption of the reading frame. The NEMO-∆ex5 protein could be observed in patient lysates following a longer period of separation (**C**). GAPDH was used as loading control, whereas bands resulting from non-specified proteins contained in the wheat germ extract were detected for in-vitro synthesized NEMO isoforms. Shown are representative western blots obtained from 3 experiments. In addition, NEMO expression was investigated in T, B, NK lymphocytes and monocytes by flow cytometry using abNEMO^150–300^ (left panel, blue histograms) and abNEMO^278–396^(right panel, red histograms) combined with anti-human CD3, CD19, CD56, and CD14 surface antibodies (**D**). Alexa fluor 488 anti-rabbit, and anti-mouse, respectively, were used as secondary antibodies and background controls (isotypes, grey histograms). In contrast to the controls, two peaks of intensity were observed in peripheral blood cell subsets obtained from the patient and her mother, which were quantified by gating

Analysis of NEMO expression in PBMC lysates revealed that no truncated protein was expressed by the patient or her mother (Fig. 3A). Whereas NEMO-∆ex4-6 protein could not be detected in any sample (Fig. 3B), NEMO-Δex5 was observed in patient lysates after prolonged separation up to the 37 kDa size marker (Fig. 3C). Full-length NEMO was expressed by all individuals tested.

In summary, NEMO-∆ex5 expression could be detected in patient blood cells by abNEMO^278–396^, but not in cells obtained from her mother or controls.

### NEMO and NEMO-Δex5 can be Discriminated as NEMO^bright^ and NEMO^dim^ Populations in Peripheral Blood cell Subsets

NEMO expression was studied in T, B, and NK lymphocytes, and monocytes by flow cytometry using abNEMO^278–396^ and abNEMO^150–300^. Cell subsets were identified by surface markers gated as shown in Figure S2A. In contrast to healthy controls, two populations expressing NEMO^bright^ and NEMO^dim^ could be detected by abNEMO^278–396^ in patient cells, and to a lower extend in her mother’s monocytes (Fig. 3D; Figure S2B). In contrast, only NEMO^bright^ expression could be found in cellular subsets of controls. The NEMO^dim^ population could not be detected by abNEMO^150–300^. This investigation was performed 6 times leading to similar results, and twice in a different laboratory that included the analysis of granulocytes (Figure S2B). Interestingly, the distribution of NEMO^bright^ and NEMO^dim^ populations among cellular subsets of the patient remained stable over time. We observed a NEMO^bright^:NEMO^dim^ ratio of approximately 75%:25% in T and B cells, an almost even (50%:50%) distribution in NK lymphocytes, and a ratio of 20%:80% in monocytes and granulocytes. A NEMO^bright^:NEMO^dim^ ratio of approximately 90%:10% was repeatedly found in the mother’s monocytes.

To compare the mean fluorescence intensities (MFIs) of full-length (WT) and Δex5 NEMO proteins, respectively, pcDNA6 vectors containing cDNAs of these variants were transfected into HCT116 colon carcinoma cells. Besides endogenous NEMO in non-transfected cells, cells transfected with WT, Δex5, or both variants were investigated for NEMO expression using abNEMO^150–300^ and abNEMO^278–396^ by flow cytometry. Consistent with results obtained by western blotting, NEMO-Δex5 was not detected by abNEMO^150–300^ but abNEMO^278–396^ (Figure S2C). However, differences between MFIs of NEMO-Δex5 and NEMO-WT proteins did not reach significance in three independent experiments (Figure S2D).

To investigate *IKBKG* variants transcribed in these populations, RNA was isolated from sorted NEMO^bright^ and NEMO^dim^ populations obtained from patient PBMCs. Both full-length and ∆ex5 transcripts were amplified by RT-PCR (Figure S2E) as confirmed by Sanger sequencing. Since analyzed by end-point PCR, the number of transcripts could not be quantified.

### Response to TNF-stimulation is Severely Diminished, Whereas Response to TLR Agonists is Mostly Retained in Peripheral Blood Cells

**Fig. 4:**
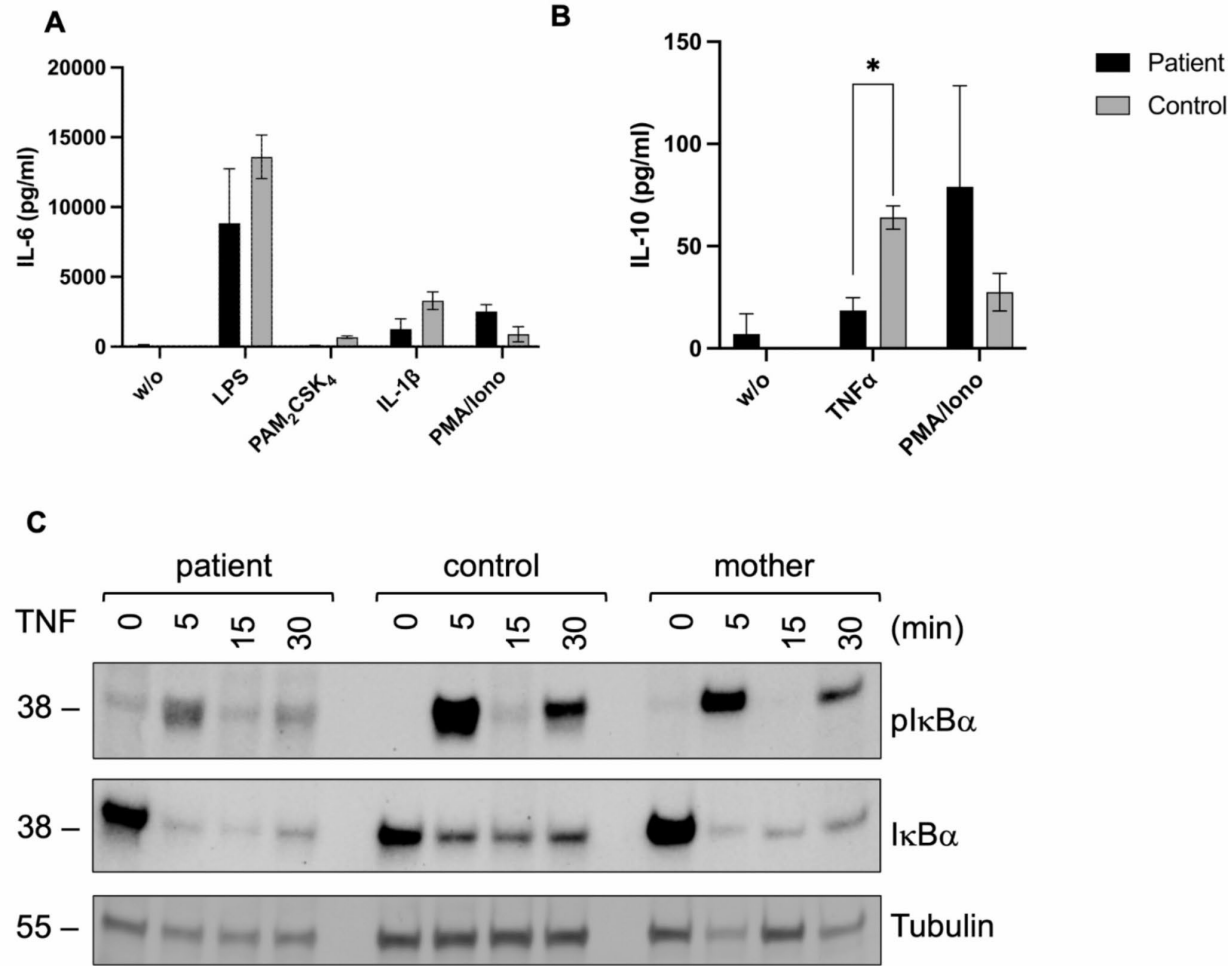
NEMO signaling function is partially impaired. Functional studies were performed on whole blood obtained from the patient and compared to shipping controls. Expression of IL-6 was measured in the supernatant following stimulation with LPS, PAM_2_CSK_2_, IL-1β and PMA/ionomycin (**A**). Levels of IL-10 were investigated after stimulation with TNF and PMA/ionomycin (**B**). Shown are the mean results with standard deviations obtained from two independent investigations. Statistics were calculated using unpaired student’s t test. PBMCs isolated from the patient and a healthy control were stimulated with TNF (200ng/ml) for 0, 5, 15, and 30 min. Levels of pIκBα and IκBα were investigated by western blotting (**C**). Protein sizes are marked on the left-hand side. Tubulin served as loading control

NEMO function was investigated in whole blood obtained from the patient and her mother by quantification of IL-6 expression in response to stimulation with the TLR4-agonist LPS, the TLR2/6-agonist PAM_2_CSK_4_, IL-1β, and PMA/ionomycin (Fig. 4A), and IL-10 expression in response to TNF and PMA/ionomycin (Fig. 4B). Whereas the response to LPS was similar in patient and controls, the TNF response was diminished in patient cells compared to healthy controls. This assay was performed twice with similar results. The mother also showed slightly impaired responses upon stimulation with LPS and TNF as compared to reference values (Table S6).

According to these findings, activation of NF-κB signaling in PBMCs upon TNF-stimulation showed reduced phosphorylation of IκBα (pIκBα) in the patient as compared to healthy controls (only one control shown) (Fig. 4C). Interestingly, pIκBα in response to TNF-stimulation was also reduced in PBMCs of her mother. Both, patient and mother, showed increased basal levels of IκBα. Of note, the patient was under treatment with TNF inhibitors at the time this investigation was performed.

Together these studies show an abrogated NEMO function with diminished response to TNF, but mostly retained TLR signaling in patient blood cells.

### NEMO-Δex5 Results in Elevated IL-18 and CXCL10 Concentrations

**Fig. 5:**
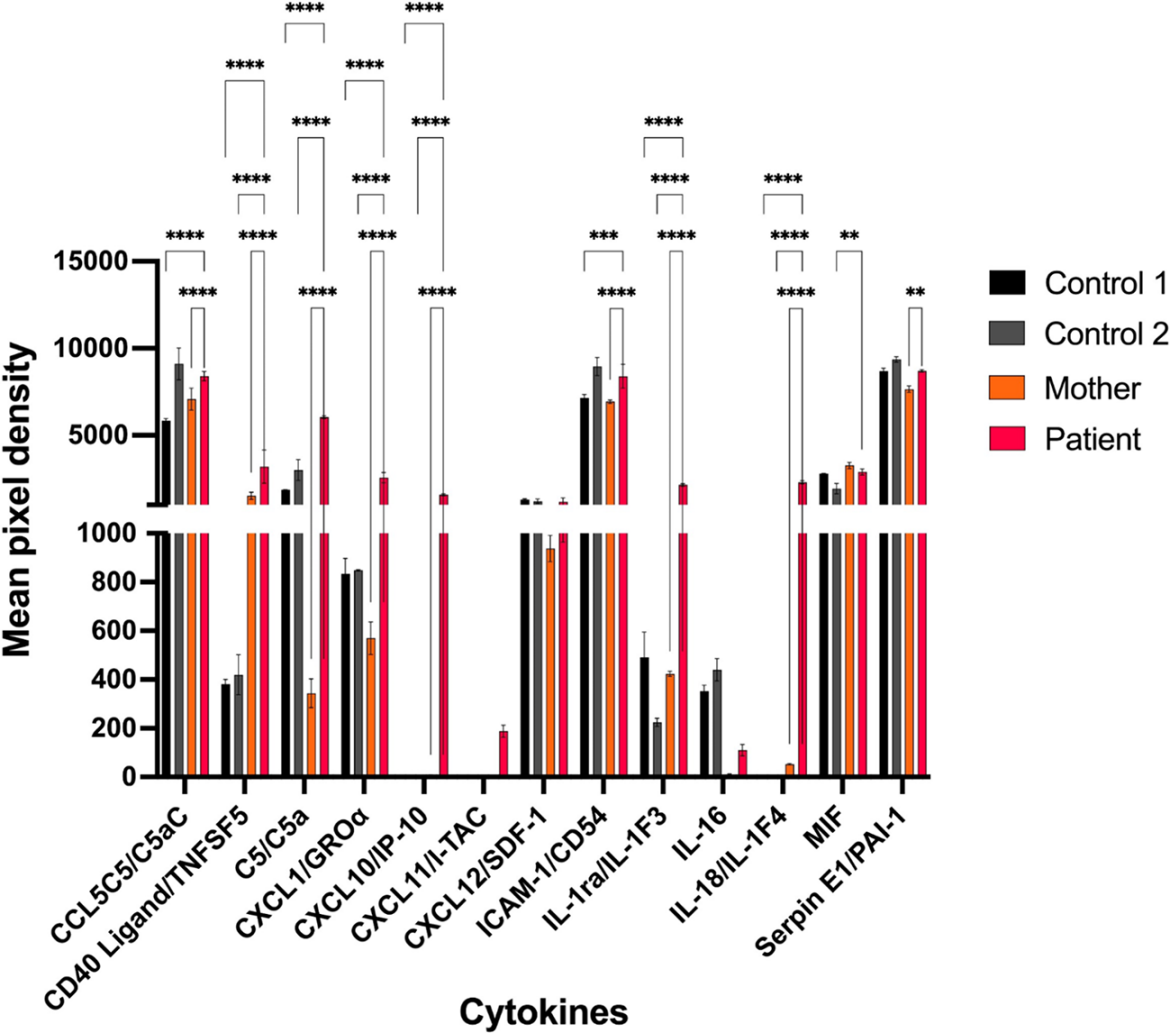
NEMO-NDAS results in elevation of cytokines and chemokines. Levels of 36 cytokines were investigated in serum obtained from the patient, her mother, and control 1 and 2 using the Human Proteome Profiler Human Cytokine Array Kit (R&D Systems). Pixel densities were analyzed by optical densitometry and normalized on included background proteins. Shown are mean densities of 13 detectable cytokines. Statistics were analyzed using two-way Anova (* *p* ≤ 0,05, ** *p* ≤ 0,01, *** *p* ≤ 0,001, **** *p* ≤ 0,0001)

Since elevated cytokines have been observed in NEMO-NDAS patients, we measured the expression of 36 cytokines/chemokines in serum samples obtained from the patient, her mother and two controls. Patient samples were collected before start of treatment with anti-TNF agents. Expression of 13 cytokines could be detected on the membrane (Figure S3) represented by two spots per protein. Spot intensities were quantified by optical densitometry and normalized by an averaged background subtraction (Table S7). CCL5C5/C5aC, CD40L/TNFSF5, C5/C5a, CXCL1/GROα, CXCL10/IP-10, ICAM-1/CD54, IL-1ra/IL-1F3, IL-18/IL-1F4, MIF, Serpin E1/PAI-1 were significantly elevated in patient serum compared to mother and controls, of which CXCL10 and IL-18 were almost exclusively expressed in the patient (Fig. 5). An increased expression of the NF-κB-dependent chemokine CXCL10 has been reported before in NEMO-NDAS 21].

Levels of total IL-18 were further investigated in patient serum samples collected during and between flares before start of treatment, as well as under treatment with adalimumab, and were compared to her mother and two controls. IL-18 level were elevated in patient samples at all time points (Table 1).

**Table 1:** IL-18 cytokine level in serum samples

Sample	Clinical presentation at time of investigation	IL-18 (pg/ml)
Patient	Fever	22.088
Patient	w/o fever	106.992
Patient	w/o fever under treatment with adalimumab	18.422
Mother	n.a.	19.465
Control 1	n.a.	n.a.

### Expression of NEMO-Δex5 is Caused by Random X Chromosome Inactivation

**Fig. 6:**
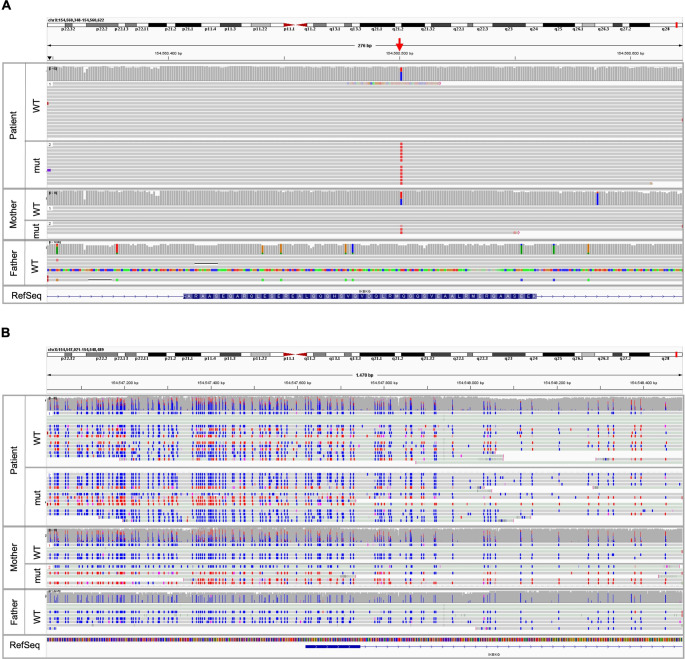
The DNA methylation status of the *IKBKG *transcription start / promoter region is skewed to one X allele in the mother, but non-skewed in the patient (**A**) Exon 5 of *IKBKG* (chrX:154,560,348 − 154,560,622 in hg38) in patient, mother and father was analyzed by Oxford Nanopore Technologies (ONT) sequencing and results are displayed in the Integrative Genomics Viewer (IGV). The *IKBKG* variant c.613 C > T p.(Gln205*) is indicated by the red arrow and was found in the mother and the patient. (**B**) The DNA methylation pattern of patient, mother and father in the transcription start / promotor region of *IKBKG* (chrX:154,547,021–154,548,489 in hg38) was analyzed by ONT sequencing and is displayed in the IGV viewer. Wildtype (WT) allele and mutated (mut) allele of the patient (top panel) show similar DNA methylation patterns, whereas the WT allele from the mother is predominantly unmethylated (blue, middle panel). The mutated allele from the mother shows a DNA methylation of ~ 60%. The WT allele from the father (lower panel) is completely unmethylated (blue). No methylation = blue, methylation = red

The DNA methylation status of the X chromosomal *IKBKG* gene was studied in genomic DNA isolated from whole blood of the patient and both parents using nanopore sequencing (Fig. 6). In the mother, the allele carrying the *IKBKG* variant was methylated to 60% around the transcription start site, whereas no DNA methylation was detected within this region at the wildtype *IKBKG* allele. Thus, the results of the mother were in the technical range for assuming skewed X-inactivation. In contrast, both X alleles of the patient were methylated to approximately 50%/50% suggesting a random X-inactivation status at this locus (Fig. 6). Similar DNA methylation patterns could be observed in regions adjacent to *IKBKG* (Figure S4).

A reversion mosaicism of the mutation in cellular subsets, as reported in several cases of NEMO deficiency [25], was excluded by Sanger sequencing (Figure S5).

## Discussion

NEMO-NDAS was recently described as an autoinflammatory syndrome caused by an increased proportion of NEMO-∆ex5 protein due to alternative splicing of *IKBKG*. The patients reported so far carried *IKBKG* splice-site variants and presented with autoinflammation including panniculitis, uveitis, hepatitis, lymphoproliferation, and cytopenia [21–23, 26]. Although most patients developed B cell lymphopenia and hypogammaglobulinemia, none had a history of severe infections or colitis. Of note, alternative splicing with exon 5-skipping has been observed in NEMO-deficient patients presenting with immunodeficiency [20, 27–29], suggesting an advantage of NEMO-∆ex5 over truncated proteins. A recent study showed that NEMO-∆ex5 forms a complex with the non-canonical kinase IKKi and participates in the TBK1/IKKi pathway that is involved in sensing of viral nucleases through TLR3. This complex resists dissociation in response to TLR3 stimulation with poly(I: C) and therefore contributes to enhanced transcription of IFN-related genes [22]. Hence, affected patients present with a type I IFN signature and systemic autoinflammation.

We report the manifestation of NDAS in a young female carrying a monoallelic variant in *IKBKG* and non-skewed lyonization in her blood cells. Besides IP, she presented with recurrent fever, occasional aphthous lesions and later developed mild hepatitis, neutropenia, and alopecia. She had no history of severe infections and, besides low vaccine titers, no signs of impaired immune function. Type I IFN-regulated genes were elevated as were levels of NF-κB associated cytokines and chemokines such as CXCL10 and CD40L. NEMO-NDAS was confirmed by an increased proportion of *IKBKG*-∆ex5 transcripts and the corresponding NEMO-∆ex5 protein in expense of full-length NEMO in peripheral blood cells. The same variant observed in this patient was reported before in a different, non-related family [20] including several female members with clinical signs of inflammation. The male index patient in this family presented with multiple episodes of severe bacterial infections besides inflammatory bowel disease. Of note, only NEMO-∆ex5, but no full-length protein was observed in his blood cells suggesting a favorable expression of this transcript variant. The patient reported here abnormally expressed NEMO-∆ex5 in line with non-skewed DNA methylation at this locus suggesting a normal pattern of X-inactivation. Based on the DNA methylation studies, the X-inactivation at the NEMO locus was skewed in her asymptomatic mother.

Functional studies of the canonical NF-κB pathway upon stimulation with IL-1β, TLR agonists (PAM_2_CSK_4_ and LPS), and TNF were performed in our patient’s blood cells. The corresponding cytokine response was more impaired upon TNF, yet only mildly affected by stimulation with IL-1β and TLR-agonists, compared to healthy controls. The predominantly diminished response to TNF compared to TLR-agonists has been reported before in NEMO-deficient patients [12, 17]. However, these findings are in sharp contrast to what has been reported in NDAS patients, in whom the nuclear translocation of phosphorylated p65 was reduced in response to poly(I: C) but retained in response to TNF-stimulation [22]. The amount of NEMO full-length and NEMO-∆ex5 proteins per cell might be critical for the clinical manifestation due to the tight regulation of the NF-κB pathways. In contrast to patients with NEMO hypomorphism, NDAS patients carrying de novo splice variants or silent mutations in *IKBKG* may express full-length NEMO in sufficient amounts besides NEMO-∆ex5. Female carriers of hypomorphic *IKBKG* variants with non-skewed lyonization are at risk to produce too much NEMO-∆ex5 that they develop NDAS, and in turn too little amount of full-length protein to prevent bacterial infections. In the patient reported here, the preserved TLR function might prevent systemic bacterial infections despite abrogated TNF response.

Interestingly, two populations expressing NEMO^bright^ and NEMO^dim^ could be identified in patient blood cells by flow cytometry. Whereas a low extend of NEMO^dim^ could also be observed in her mother’s cells, only NEMO^bright^ was expressed in healthy controls. During several investigations over a period of 3 years, the ratio of NEMO^bright^:NEMO^dim^ appeared to be stable in both females. The fact that NEMO^dim^ could only be detected when using an antibody that recognizes NEMO-∆ex5, suggested either a lower antibody affinity to NEMO-∆ex5 or a reduced content of total NEMO protein in cellular subsets expressing NEMO-∆ex5. To compare MFIs between NEMO and NEMO-∆ex5 proteins, we transfected plasmids coding for full-length or ∆ex5 transcripts, respectively, into HCT116 cells. Results were normalized on endogenous NEMO expression of this cell line. NEMO-∆ex5 was detected by abNEMO^278–396^ with similar intensity as full-length NEMO was detected by abNEMO^150–300^. The possibility of lower total NEMO protein content in NEMO^dim^ populations is favored by the observation of reduced fluorescent intensity in PBMCs of male patients with hypomorphic NEMO deficiency using this assay [12].

Corresponding to the almost exclusive expression of *IKBKG-*∆ex5 transcripts determined in patient monocytes by RT-PCR, NEMO^dim^ expression was observed in more than 80% of CD14^+^ cells using flow cytometry. The ratio of WT:*∆*ex5 transcripts in T cells was around 32%:68% and in NK cells 50%:50%, excluding the other transcript variants. In B cells, *IKBKG-*∆ex4-6 instead of *IKBKG*-∆ex5 was predominantly observed, which might have technical reasons. Investigation of *IKBKG* transcripts expressed in NEMO^bright^ and NEMO^dim^ PBMCs by RT-PCR revealed that both WT and ∆ex5 transcripts were present in both populations. Since small amounts of the ∆ex5 variant transcripts can be found in healthy controls, it is reasonable that patient cells expressing the WT *IKBKG* allele contain both full-length NEMO and NEMO-∆ex5 proteins at the same time. Cells expressing the *IKBKG* variant may have lower amounts of total NEMO protein, which is predominantly NEMO-∆ex5 as detected by abNEMO^278–396^.

Although several cytokines and chemokines were elevated in patient serum samples compared to controls, CXCL10 and IL-18 could only be detected in the patient. Increased CXCL10 levels have been reported in NDAS patients [21], and may be related to enhanced NF-κB signaling [30]. Processing and release of IL-18 is mediated by NLRP1, NLRP3, NLRC4, and pyrin inflammasome-activated caspase-1 (CASP-1) [31]. Extremely high levels of IL-18 can be observed in patients affected by NLRC4 inflammasomopathies [31–33], or macrophage activation syndrome (MAS) [34]. We therefore investigated expression of total IL-18 in serum samples, which was mildly elevated in the patient at three different time points, but without correlation to flares. Since NF-κB is involved in the regulation of the inflammasome-induced release of IL-18 [35], we conclude that the mild elevation of IL-18 may result from enhanced NF-κB signaling due to higher proportions of NEMO-∆ex5.

The systemic inflammation observed in the patient reported here partially responded to treatment with anti-TNF agents, although mild hepatitis and neutropenia persisted. In concordance with the clinical presentation, the score of her IFN signature even increased. In this study, we investigated the proportion of NEMO variants in peripheral blood cells, however, altered ratios of NEMO proteins impacting on NF-κB signaling pathways may also be present in other cell types. The inflammation in NEMO hypomorphism and NEMO-NDAS is driven by increased TNF-mediated cell death [36] stimulating the production of proinflammatory cytokines by immune cells. Therefore, anti-TNF agents are a well-established therapy for inflammatory disorders caused by NEMO defects. However, TNF inhibition alone may not be able to efficiently control the disease, and alternative drugs either targeting the TNF signaling pathway, such as RIPK1 inhibitors [37], or interfering with splice variants will be needed. Hematopoietic stem cell transplantation, which is a reasonable approach in NEMO hypomorphism [38], might not be able to reduce the amount of NEMO-∆ex5 sufficiently to prevent clinical symptoms, as additional expression in epithelial cells can be expected [20, 39].

In summary, this study provides an example of the tight regulation of NF-κB pathways by different NEMO transcript variants. The patient reported here carries a monoallelic mutation in *IKBKG*, which is expressed in various ratios because of non-skewed lyonization. This leads to expression of increased amounts of NEMO-∆ex5 due to alternative splicing. The altered distribution of full-length NEMO and NEMO-∆ex5 proteins results in the clinical presentation of NDAS. Diminished quantities of full-length NEMO protein constrain TNF signaling, while preserving a competent response to TLR activation to mitigate the risk of infections.

## Electronic Supplementary Material

Below is the link to the electronic supplementary material.

Supplementary Material 1

Supplementary Material 2

Supplementary Material 3

Supplementary Material 4

Supplementary Material 5

Supplementary Material 6

## Data Availability

The datasets generated and analysed during the current study are available from the corresponding author on reasonable request.
