# Supplementary figures and images for: Non-Skewed X-inactivation Results in NF-κB Essential Modulator (NEMO) Δ-exon 5-autoinflammatory Syndrome (NEMO-NDAS) in a Female with Incontinentia Pigmenti
Source: J Clin Immunol. 2024 Sep 12;45(1):1. doi: 10.1007/s10875-024-01799-2 (PMC11393190; doi:10.1007/s10875-024-01799-2)

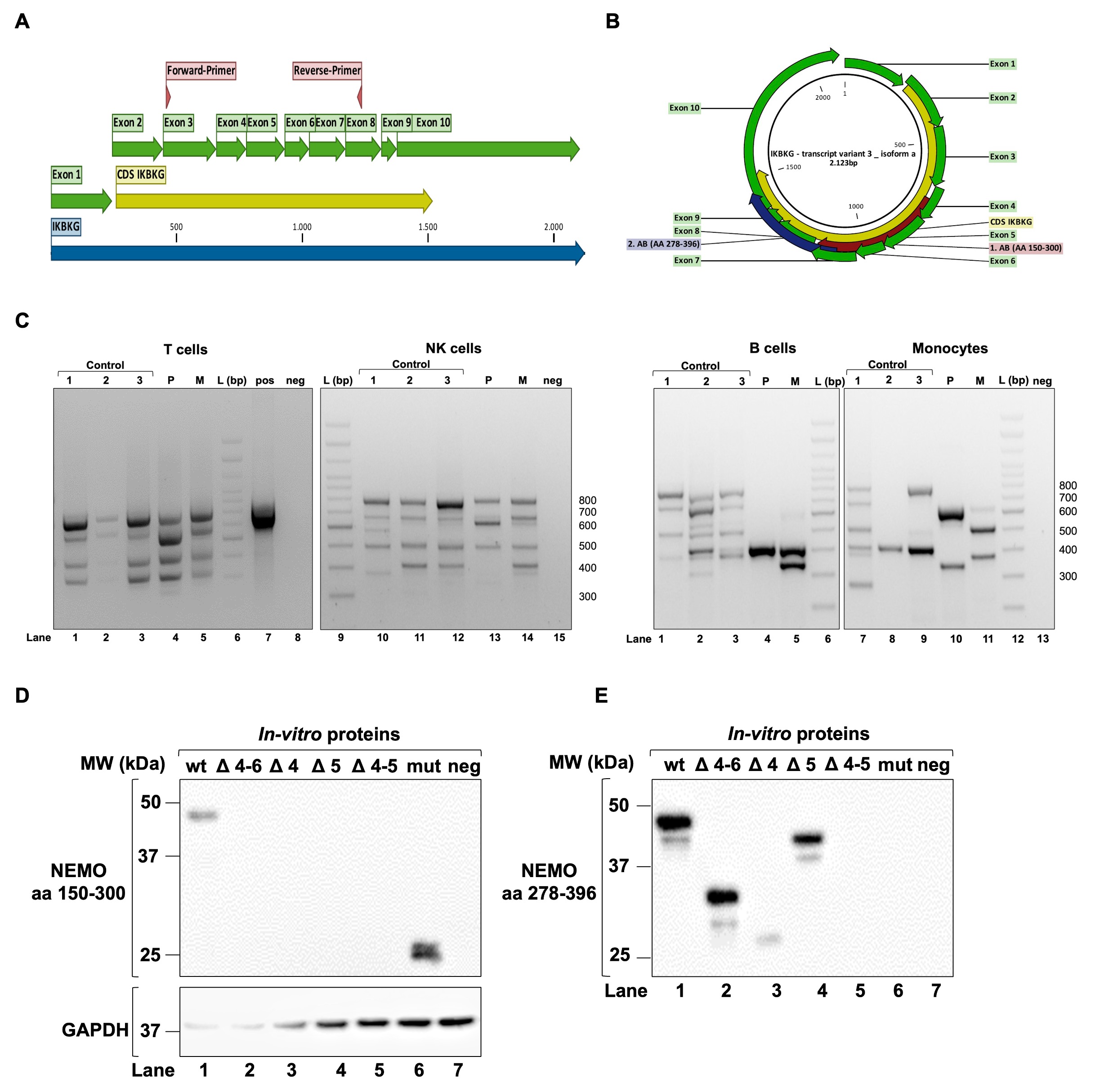

Supplement: Supplementary file 1 — Supplementary Material 1 [file 10875_2024_1799_MOESM1_ESM.jpg]

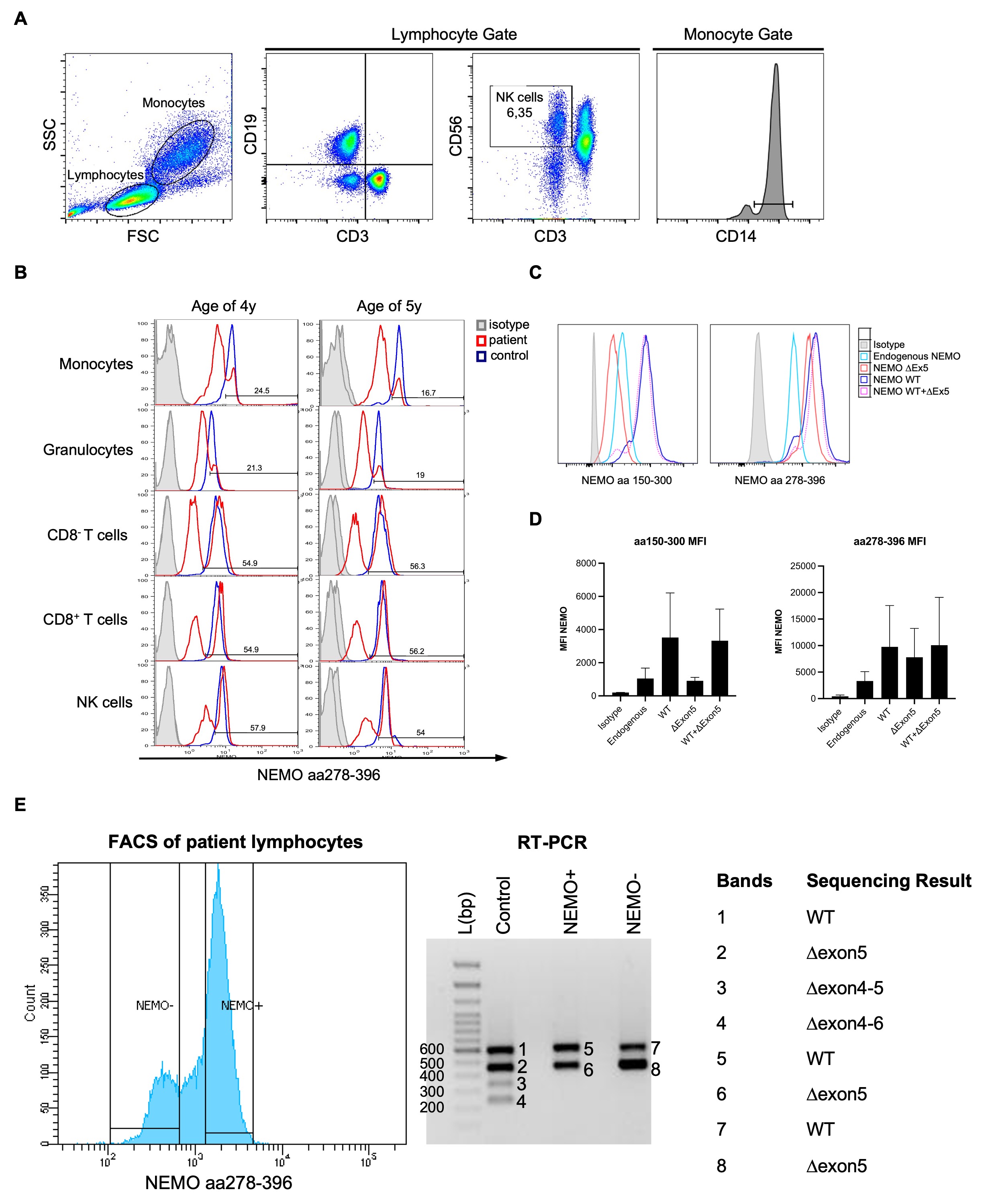

Supplement: Supplementary file 2 — Supplementary Material 2 [file 10875_2024_1799_MOESM2_ESM.jpg]

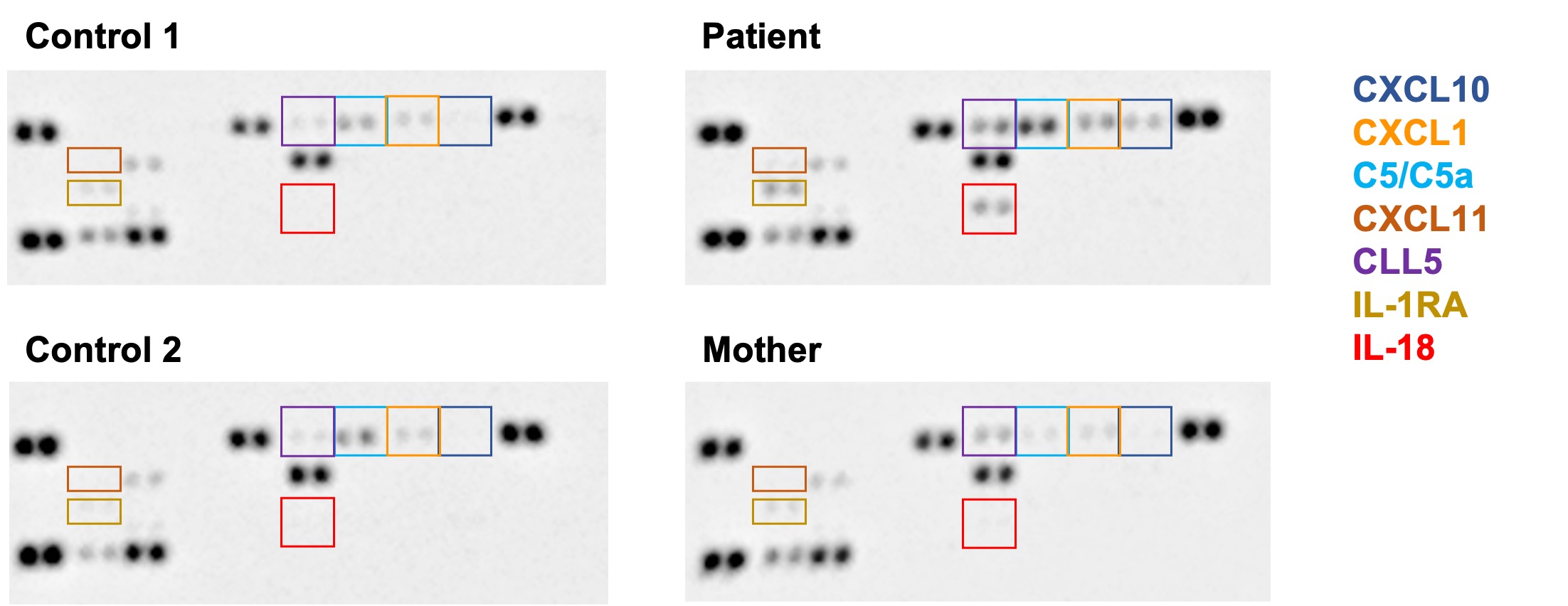

Supplement: Supplementary file 3 — Supplementary Material 3 [file 10875_2024_1799_MOESM3_ESM.jpg]

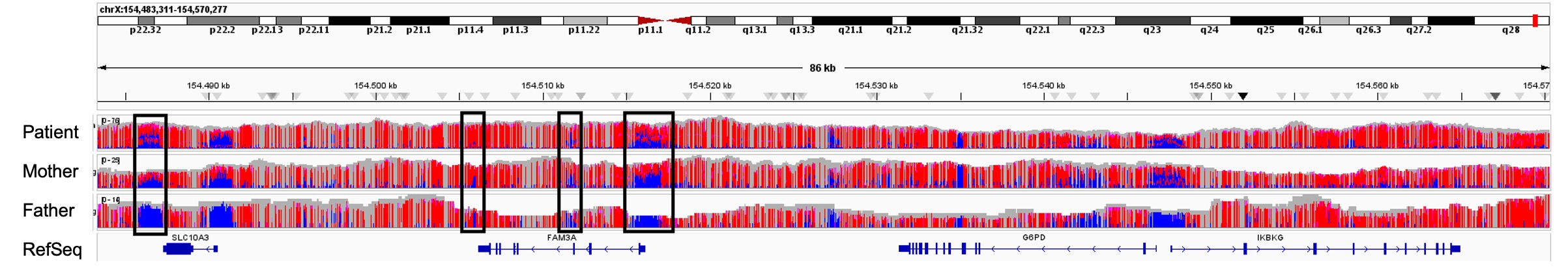

Supplement: Supplementary file 4 — Supplementary Material 4 [file 10875_2024_1799_MOESM4_ESM.jpg]

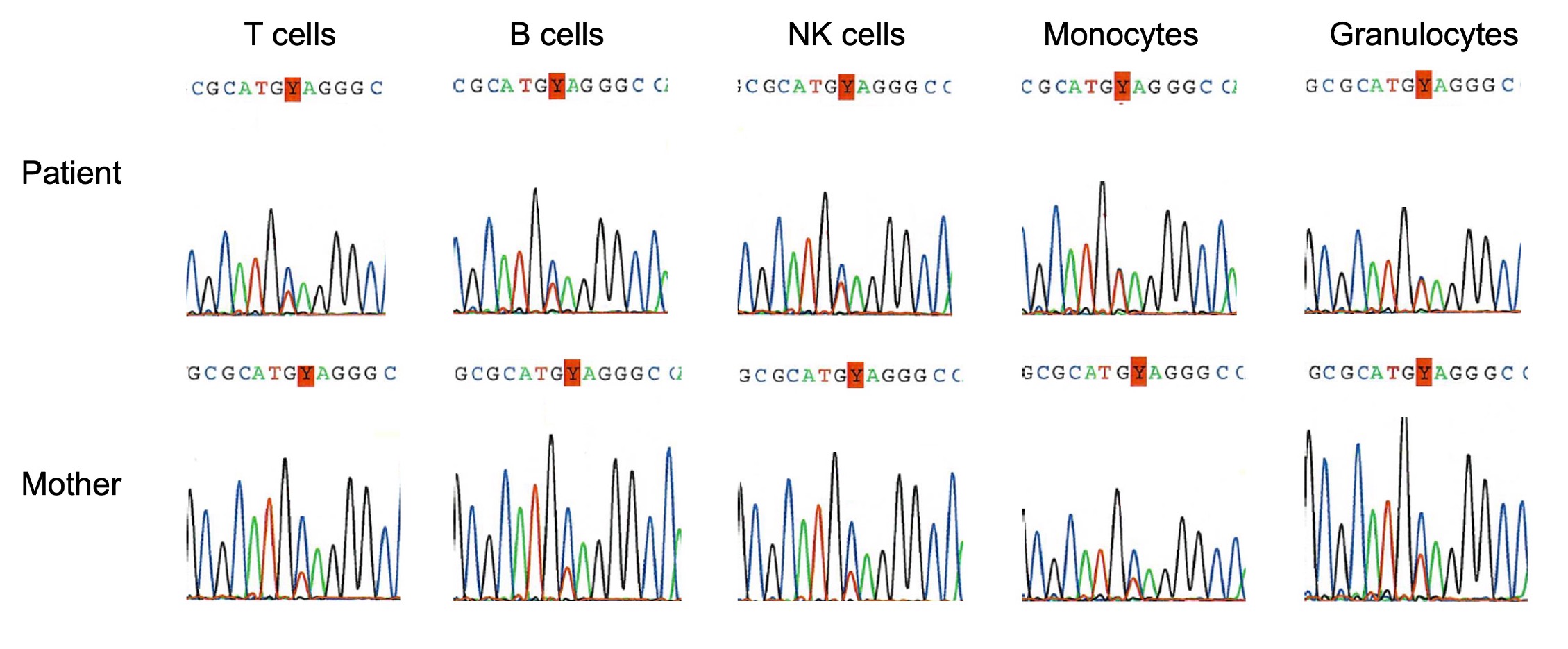

Supplement: Supplementary file 5 — Supplementary Material 5 [file 10875_2024_1799_MOESM5_ESM.jpg]
