# Supplementary material for: Non-Skewed X-inactivation Results in NF-κB Essential Modulator (NEMO) Δ-exon 5-autoinflammatory Syndrome (NEMO-NDAS) in a Female with Incontinentia Pigmenti
Source: J Clin Immunol. 2024 Sep 12;45(1):1. doi: 10.1007/s10875-024-01799-2 (PMC11393190; doi:10.1007/s10875-024-01799-2)
Supplement: Supplementary file 6 — Supplementary Material 6 [file 10875_2024_1799_MOESM6_ESM.docx]

**Supplementary Material**

**Supplementary Methods and Materials**

**RT-PCR**

*IKBKG* was amplified from cDNA using primers spanning exon 3-8, or 3-7, respectively, (**Table S1**) and HotStarTaq polymerase (Qiagen). PCR products were loaded onto 1-2% (w/v) TAE buffered (Rotiphorese® TAE buffer, Roth) agarose gels (Sigma-Aldrich) and run for 40-60min at 85V (<1kb), or 60-90min at 70-80V (>1kb), respectively. 1 kb (VWR chemiclas) and 100 bp (Thermo Fisher Scientific) ladders were loaded for size determination.

**Table S1: Primers**

| **Gene** | **Forward/**  **Reverse** | **Sequence 5’-3’** |
| --- | --- | --- |
| **Quality control cDNA synthesis** | | |
| *ACTB* (Beta actin) | F | CCA ACA CAG TGC TGT CTG |
|  | R | CAA CTA AGT CAT AGT CCG CC |
| **RT-PCR *IKBKG* transcripts** | | |
| *IKBKG ex 3-8* | F | AGA GCA ACC AGA TTC TGC GG |
|  | R | CCT TCT TCT CGG CCA GCT TC |
| *IKBKG ex 3-7* | F | AGA GCA ACC AGA TTC TGC GG |
|  | R | TGC TGG AGC TGC TGT TTG AG |
| **Cloning** | | |
| *IKBKG* ex2-10-KpnI | F | GGG GTA CCA CCA TGA ATA GGC ACC TCT GGA AG |
| *IKBKG* ex2-10-NotI | R | ATA AGA ATG CGG CCG CCT ACT CAA TGC ACT CCA TGA C |
| **Sequencing plasmids** | | |
| Seq-insert-pcDNA6 | F | GCT AAC TAG AGA ACC CAC TGC |
|  | R | GCT GAT CAG CGG GTT TAA ACT |
| pCR 2.1 TOPO | F | GTA AAA CGA CGG CCA G |
|  | R | CAG GAA ACA GCT ATG AC |

**Sanger sequencing**

Transcript variants obtained from RT-PCR were sequenced using Sanger sequencing.

PCR products were purified by gel electrophoresis, and DNA of fragments was isolated by gel extraction (peqGold). Samples were amplified using the BigDye Terminator v1.1 Cycle Sequencing Kit (Thermo Fisher Scientific) and appropriate forward or reverse primers. Sequencing was performed on a 3130 Genetic Analyzer (Applied Biosystems).

**Oxford Nanopore Sequencing of *IKBKG* transcripts and data analysis**

Nanopore sequencing (Oxford Nanopore Technologies (ONT)) was performed in two separate runs on RT-PCR products obtained from whole PBMCs and sorted T, B, NK lymphocytes and monocytes.

RT-PCR products were purified using the QIAquick PCR Purification Kit (Qiagen). Two PCR products obtained from sorted cells were pooled to increase the DNA content. Samples were quantified using the Qubit dsDNA BR Assay (Thermo Fisher Scientific) on a POLARstar OMEGA Microplate Reader (BMG Labtech). For library preparation, the Native Barcoding Kit (ONT) was used according to the manufacturer protocol. 0.1 pmol of the library was sequenced on a Flongle (R9) flow cell, which was placed into a MinION sequencing device (ONT). Runs were conducted using MinKNOW software. Raw data was basecalled and demultiplexed using Guppy software version 4.0.14 (ONT) with a barcode-threshold of 80%.

The data was imported into CLC Genomics Workbench and barcodes as well as adapters were trimmed off the reads. Data was size selected and mapped against hg19 (GenBank assembly accession: GCA_000001405.1), respectively *IKBKG* (accession no. NG_009896) using minimap2 [1]. Subsequently, an *IKBKG* transcript library (**Table S2**) was created, and data was examined for unique transcript variants. In total, 51 *IKBKG* transcript variants could be identified, of which 7 had been published in the NCBI GenBank (https://www.ncbi.nlm.nih.gov/gene/8517) databank before. Another minimap2 alignment step and an RNA-Seq-Analysis in CLC Genomics was performed using the created *IKBKG* transcript library as reference. Aligned reads to the corresponding transcript variants of the reference sequence were counted. Transcript variants below the threshold of 5 % of all reads in all five subjects were excluded from further analysis.

**Table S2:** ***IKBKG* transcript variant library**

| **Name** | **Region** |
| --- | --- |
| **NCBI references** | |
| NM_001099857.5 | Exon 2-10 |
| NM_001099856.6 | Exon 2-10* |
| NM_001145255.4 | Exon 2-4, 6, 8-10 |
| NM_001321397.3 | Exon 2-3, 4**,5-10 |
| NM_001377314.1 | Exon 2-3, 4**, 6-10 |
| NM_001377315.1 | Exon 2-3, 7-10 |
| NR_165197.1 | Exon 2-3, 6-10 |
| **Used transcript variant references (seen in transcript library after amplification of exon 3 – 8)** | |
| Transcript variant 1 | Exon 3 (109 bp) - exon 7 (41 bp) - exon 8 complete |
| Transcript variant 2 | Exon 3 (81 bp) - exon 7 (41 bp) - exon 8 complete |
| Transcript variant 3 | Exon 3 (100 bp) - exon 7 (41 bp) - exon 8 complete |
| Transcript variant 4 | Exon 3 (81 bp) - exon 7 (77 bp) - exon 8 complete |
| Transcript variant 5 | Exon 3 (94 bp) - exon 7 (107 bp) - exon 8 complete |
| Transcript variant 6 | Exon 3 (91 bp) - exon 7 (107 bp) - exon 8 complete  Transcript variant 12 Exon 3, 6, 8 complete - exon 7 (78 bp) |
| Transcript variant 7 | Exon 3 (81 bp) - exon 7 (116 bp) - exon 8 complete |
| Transcript variant 8 | Exon 3 (87 bp) - exon 7 (115 bp) - exon 8 complete |
| Transcript variant 9 | Exon 3 (109 bp) - exon 7 (77 bp) - exon 8 complete |
| Transcript variant 10 | Exon 3 (87 bp) - exon 7 (116 bp) - exon 8 complete |
| Transcript variant 11 | Exon 3, 7-8 complete |
| Transcript variant 12 | Exon 3, 6, 8 complete - exon 7 (78 bp) |
| Transcript variant 13 | Exon 3, 6-8 complete |
| Transcript variant 14 | Exon 3, 5-8 complete |
| Transcript variant 15 | Exon 3, 6-8 complete - exon 5 (68 bp) |
| Transcript variant 16 | Exon 3, 6, 8 complete - exon 5 (55 bp) - exon 7 (135 bp) |
| Transcript variant 17 | Exon 3-4, 6-8 complete |
| Transcript variant 18 | Exon 3-4, 6, 8 complete - exon 7 (135 bp) |
| Transcript variant 19 | Exon 3, 5-6, 8 complete - exon 7 (135 bp) |
| Transcript variant 20 | Exon 3-4, 6-7 complete - exon 5 (90 bp) - exon 8 (39 bp) |
| Transcript variant 21 | Exon 3-4, 6-8 complete - exon 5 (69 bp) |
| Transcript variant 22 | Exon 3-4, 6-8 complete - exon 5 (split 86 bp) |
| Transcript variant 23 | Exon 3-5, 7-8 complete |
| Transcript variant 24 | Exon 3-4, 6-8 complete, exon 5 (144 bp) |
| Transcript variant 25 | Exon 3-4, 6-8 complete - exon 5 (130 bp) |
| Transcript variant 26 | Exon 3 complete - exon 8 (27 bp) |
| Transcript variant 27 | Exon 3-4 complete - exon 8 (23 bp) |
| Transcript variant 28 | Exon 3, 8 complete - exon 7 (66 bp) |
| Transcript variant 29 | Exon 3-6, 8 complete - exon 7 (134 bp) |
| Transcript variant 30 | Exon 3, 5-8 complete - exon 4 (106 bp) |
| Transcript variant 31 | Exon 3 (109 bp) - exon 7-8 complete |
| Transcript variant 32 | Exon 3 (116 bp) - exon 7-8 complete |
| Transcript variant 33 | Exon 3, 8 complete |
| Transcript variant 34 | Exon 3-5 complete - exon 8 (23 bp) |
| Transcript variant 35 | Exon 3, 7-8 complete - exon 4 (109 bp) - exon 6 (88 bp) |
| Transcript variant 36 | Exon 3, 8 complete - exon 4 (73 bp) |
| Transcript variant 37 | Exon 3-5, 7-8 complete - exon 6 (66 bp) |
| Transcript variant 38 | Exon 3, 5-8 complete - exon 4 (35 bp) |
| Transcript variant 39 | Exon 3-5, 8 complete - exon 7 (77 bp) |
| Transcript variant 40 | Exon 3-6, 8 complete - exon 7 (128 bp) |
| Transcript variant 41 | Exon 3-4 complete - exon 8 (27 bp) |
| Transcript variant 42 | Exon 3 (53 bp) - exon 5 (130 bp) - exon 6-8 complete |
| Transcript variant 43 | Exon 3, 5-8 complete - exon 4 (109 bp) |
| Transcript variant 44 | Exon 3, 5-8 complete - exon 4 (116 bp) |

*uses an alternate promoter and initiates translation from an alternate in-frame upstream start codon compared to variant NM_001099857.5

**exon 4: start codon CAG missing

**Oxford Nanopore Sequencing and library preparation for X-inactivation studies**

For methylation analysis and variant phasing, nanopore sequencing with adaptive sampling for the patient and whole genome sequencing from father and mother and 5hmC and 5mC methylation calling was conducted. For this purpose, genomic DNA was extracted using the FlexiGene DNA Kit (250) (Quiagen, #51206), according to the manufacturer´s manual. The DNA was dissolved overnight at 4°C and quantified by using the Nanodrop (ThermoFisher Scientific). 9 µg of the extracted DNA was size selected using SRE Kit (PacBio, #204053) and 3 µg were used as input for nanopore library preparation with the ligation sequencing DNA V14 kit (ONT, SQK-LSK114) according to the manufacturer´s protocol. Each library was loaded on a Promethion R10.4.1 flow cell (ONT, FLO-PRO114M) and sequenced with a P2Solo device for up to 72 h. Throughout the sequencing process, the flow cells were reloaded 1-2 times after a nuclease flush (ONT, EXP-WSH004) with the same library according to the manufacturer´s instructions, to enhance sequencing yield. For adaptive sampling chromsome X (chrX: 1- 156,040,895) of the hg38 genome with the “fast modified basecalling for 5hmC and 5mC in CG context” basecall model of the MinKNOW software version 23.11.7 was set.

Analysis, phasing and methylation

The Bamfiles from mother, father and patient were subjected to analysis. The Epi2Me human variation workflow (version 1.9.0, available at <https://github.com/epi2me-labs/wf-human-variation>) was executed to generate DNA methylation data, structural variant data, and phased aligned BAM files (based on the GRCh38 reference genome) for the genomic region spanning coordinates of whole chromosome X. A minimum coverage threshold of 1 was applied. Additionally, the BAM files underwent manual inspection using the Integrative Genomics Viewer (IGV, version 2.17.0).

**Cloning**

The WT, Dex5, Dex4, Dex4-5, and Dex4-6 *IKBKG* transcript variants were cloned into the pcDNA6 vector under the T7 promoter to be subsequently used for the *in-vitro* synthesis of proteins. The full-length *IKBKG* transcript, the c.613C>T variant, and the Δex5 variant had already been cloned into pcDNA4 plasmids (**Table S3**) for a previous study [2], while the remaining transcript variants Δex4, Δex4-5, Δex4-6 were amplified from human cDNA. In a first step, variants were amplified from their templates using Phusion Hot Start Polymerase (Thermo Fisher Scientific). For the subsequent restriction digests, corresponding restriction sites were added to pcDNA4-derived products using the respective primer pairs (**Table S1**). PCR products amplified from plasmids were purified using the peqGold Cycle Pure Kit (Peqlab), products amplified from cDNA were separated by gel electrophoresis on a 2% TE-buffered gel and excised bands were purified using the peqGold Gel Extraction Kit (Peqlab).

To facilitate cloning into the pcDNA6 vector with sufficient material, *IKBKG* transcripts amplified from human cDNA were first cloned into the TOPO TA vector system using the TOPO^TM^ TA Cloning^TM^ Kit (Thermo Fisher Scientific) and XL2-Blue Ultracompetent cells (Agilent) for transformation.

At a later stage, cDNA-derived variants were digested together with the newly created pcDNA6 NEMO-wild-type vector as backbone using the restriction enzymes *Sbf*I-HF and *Xcm*I (New England Biolabs). DNA concentrations were determined on a NanoDrop 8000 (Thermo Fisher Scientific).

The Fast-Link^TM^ DNA Ligation Kit (Lucigen) was used for ligation of vector backbones and inserts according to the manufacturer’s instructions (except for overnight ligation at RT and subsequent deactivation at 70°C for 15min).

Cloning products were transformed into XL2-Blue Ultracompetent cells (Agilent) and grown on LB agar plates containing 200µg/ml ampicillin. Picked colonies were screened for successful insert integration by Taq polymerase PCR (Qiagen). Plasmid DNA was purified using the QIAprep Spin Miniprep Kit (Qiagen) and BioSprint 15 (Qiagen). Correct sequences were confirmed by Sanger sequencing.

All vectors containing *IKBKG* transcript variants were linearized by PmeI-digest at 37°C overnight and extracted by precipitation with phenol-chloroform and ethanol. 1μg of template was used for protein synthesis using the TNT T7 Coupled Wheat Germ Extract System (Promega).

**Table S3: Plasmids**

| **Name** | **Acronym** | **Cloning-site: *IKBKG* exon** |
| --- | --- | --- |
| **Plasmids used as templates** | | |
| pcDNA6 myc-His A | pcDNA6 Vector |  |
| pcDNA4 NEMO-A-Wt K1 [ERJ3170] | pcDNA4-Wt | 2-3-4-5-6-7-8-9-10 |
| pcDNA4 NEMO-A-Mut K2 [ERJ7315] | pcDNA4-Mut | 2-3-4-5*-6-7-8-9-10 |
| pcDNA4 NEMO-C K1 [ERJ7315] | pcDNA4-Neg | 2-3-4-6-7-8-9-10 |
| **Plasmids generated** | | |
| pcDNA6 NEMO-wild-type | NEMO wt | 2-3-4-5-6-7-8-9-10 |
| pcDNA6 NEMO-mutant | NEMO mut | 2-3-4-5*-6-7-8-9-10 |
| pcDNA6 NEMO deleted exon 5 | NEMO Δexon5 | 2-3-4-6-7-8-9-10 |
| pcDNA6 NEMO deleted exon 4 | NEMO Δexon4 | 2-3-5-6-7-8-9-10 |
| pcDNA6 NEMO deleted exon 4-5 | NEMO Δexon4-5 | 2-3-6-7-8-9-10 |
| pcDNA6 NEMO deleted exon 4-6 | NEMO Δexon4-6 | 2-3-7-8-9-10 |

* exon 5 containing the point mutation c.613C>T

**Western Blotting of NEMO proteins**

PBMCs were lysed in 100μl lysis buffer (50mM Tris-HCl, 62.5 mM EDTA, 1% Nonidet P40, 0.4% DOC, and 1x tablet of protease inhibitor (Roche)) on ice for 30min. Protein content was quantified using the DC Protein Assay (BioRad) measured on the POLARstar OMEGA Microplate Reader (BMG Labtech).

15μg protein of samples, or 2-5μl of in-vitro synthesized proteins, respectively, were boiled at 95°C for 5min in 4x Laemmli sample buffer (BioRad) and loaded onto hand casted 10% SDS-PAGE gels (Bio-Rad). The PageRulerTM Plus Prestainded Protein Ladder (10-250kDa) (Thermo Fisher Scientific) was used for size determination. Gelelectrophoresis was performed in Rothiphorese® SDS-PAGE running buffer (Roth) for 20min at 120V, subsequently increased to 180 V. Proteins were transferred onto a PVDF membrane using transfer buffer (48mM Tris, 39mM Glycin, 2% Methanol) and the TE 77 Semi-Dry Transfer Unit (Cytiva Amersham^TM^) at 65mA for 1h 20min. Membranes were blocked in 5% milk powder dissolved in 1x TBST buffer (100mM Tris pH 7.2-8.0, 1.5M NaCl, 0.5 % Tween 20) for 1h at RT. After blocking, membranes were incubated with primary antibodies (Recombinant rabbit anti-IKK gamma/NEMO (ab188569, Abcam) 1:10000; purified mouse anti-IKK gamma/NEMO (611306, BD) 1:1000; rabbit anti-GAPDH (ab181602, Abcam) 1:40000) overnight at 4°C. On the next day, membranes were washed three times in 1x TBST buffer (10min, RT), and incubated with secondary antibodies (Goat Anti-Rabbit IgG (H+L)-HRP conjugate (1706515, BioRad) 1:5000; Goat Anti-Mouse IgG (H+L)-HRP conjugate (1706516, BioRad) 1:3000) for 2h at RT. Membranes were developed using the SuperSignal^TM^ West Pico Chemiluminescent Substrate (Thermo Fisher Scientific) and signals were detected with a FUSION SL (Vilber Lourmat). Membranes were stripped using Restore PLUS Western Blot Stripping Buffer (Thermo Fisher Scientific) to detect the loading control GAPDH. Of note, unspecific bands were detected for *in-vitro* synthesized NEMO proteins when using the anti-GAPDH antibody, which result from proteins contained in the wheat germ extract as indicated by the manufacturer. NEMO proteins were quantified by optical densitometry with ImageJ software [3, 4] and normalized on intensities of GAPDH.

**Transfection of *IKBKG* variants into HCT116 cells**

WT and ∆ex5 *IKBKG* plasmids were linearized by digestion with PmeI (NEB) at 37°C over-night. RNA of *IKBKG* variants was transcribed using the mMESSAGE mMACHINE T7 transcription kit (Invitrogen). Synthesized RNAs were subsequently modified by polyadenylation using the Poly(A)-Tailing kit (Invitrogen) and extracted (RNAeasy mini kit; Qiagen)). Concentration of RNA was measured on the NanoDrop spectrophotometer.

3µg RNA of each WT, ∆ex5, WT+∆ex5, and GFP, respectively, were transfected into 1x10^6^ HCT116 cells in suspension using the Cell Line Nucleofector® Kit V (Lonza) and program D-32 on a Nucleofector® II (Amaxa) device. Transfected cells were cultured on a 6-well plate in McCoy medium (Gibco) at 37°C for 24h. Transfection efficiency was determined by GFP expression using flow cytometry and found to be 89-97% in all three experiments performed. Cells transfected with WT, ∆ex5, and WT+∆ex5 *IKBKG* RNA, as well as an electroporated but non-transfected control, were detached from culture plates using Accutase (Thermo Fisher Scientific). Samples were permeabilized and analyzed for NEMO expression (ab150-300 and ab278-396) using flow cytometry as described below.

**Flow cytometry and cell sorting**

PBMCs were permeabilized in 100µl of solution A from the Fix & Perm^TM^ Kit (GAS004, Thermo Fisher Scientific) diluted 1:1 with PBS (Gibco^TM^, Thermo Fisher Scientific) (15min, RT), washed twice using 1x Perm/Wash solution (BD) and blocked with 10% goat serum (Sigma-Aldrich) (30min, RT). Cells were resuspended in recombinant rabbit anti-IKK gamma/NEMO (ab188569, Abcam) (1:100), or purified mouse anti-IKK gamma/NEMO (611306, BD) (1:50), respectively, and incubated 1h at RT. Following two washes with Perm/Wash, cells were stained with Alexa Fluor 488 goat anti-mouse (1:200), or Alexa Fluor goat anti-rabbit (1:100) (both Thermo Fisher Scientific), respectively, for 1h at RT in the dark. Secondary antibodies were used as background controls. After washes with Perm/Wash and PBS, cells were stained with antibodies detecting surface marker (APC-Cy7 anti-human CD3, APC anti-human CD14, PerCP anti-human CD19, PECy7 anti-human CD56 (Biolegend) 1:100 in PBS, incubated 20min at RT), washed and resuspended in PBS supplemented with 1% fetal bovine serum (FBS) (PAN-Biotech). Samples were acquired on a FACS Aria I and analyzed according to the gating strategy shown in **Figure S2A**.

T, B, NK lymphocytes, and monocytes were sorted from PBMCs on a FACS Aria I (BD) following immunofluorescent staining with PerCP/Cyanine5.5 anti-human CD3 (Biolegend), FITC anti-human CD19 (Biolegend), APC anti-human CD56 (Biolegend), PE anti-human CD14 (MP9) (BD), and APC-Cy7 anti-human CD45 (BD) in PBS supplemented with 1% FBS for 20min at RT in the dark.

**NEMO functional studies on PBMCs**

PBMCs obtained from the patient, her mother and two healthy controls were stimulated or not with 200ng/ml TNF (Peprotech) in RPMI (Gibco) supplemented with 10% FCS (Gibco), 1% L-Glutamine (Gibco) and 1% Pen/Strep (Gibco). Cells were washed twice with ice-cold PBS (Gibco) prior to lysis in lysis buffer (30 mM Tris-HCl, pH 7.4, 150 mM NaCl, 2 mM EDTA, 2 mM KCl, 10% Glycerol) supplemented with 1% Triton X-100, 1x complete EDTA-free protease-inhibitor mix (Roche), and 1x phosphatase-inhibitor cocktail 2 (Sigma Aldrich). Protein concentration was determined using BCA protein assay (Thermo Fisher Scientific). Lysates were denatured in reducing sample buffer before separation by SDS-PAGE (BoltTM 4-12% Bis-Tris, Thermo Fisher Scientific). Proteins were blotted onto membranes with the Trans-Blot Turbo Transfer System (BioRad). Membranes were incubated with primary antibodies (phospho-IκBα (9246, IgG1, Cell Signaling Technology (CST), dilution 1:1000), IκBα (9242, rabbit, CST, dilution 1:1000), Tubulin (12004166, Rhodamine-labelled, Bio-Rad, dilution 1:5000)) at 4°C overnight or for 1 hour at room temperature. Washing of membranes was performed in 1xTBS containing 0.1% Tween-20 (Sigma Aldrich) for 3 × 10 min prior to incubation with the respective HRP-conjugated secondary antibody (Southern Biotech) for 1h at RT. Membranes were subsequently imaged on a ChemiDoc Imaging System.

**Supplementary References**

1. Li H. Minimap2: pairwise alignment for nucleotide sequences. Bioinformatics. 2018;34(18):3094-100.

2. Klemann C, Pannicke U, Morris-Rosendahl DJ, Vlantis K, Rizzi M, Uhlig H, et al. Transplantation from a symptomatic carrier sister restores host defenses but does not prevent colitis in NEMO deficiency. Clin Immunol. 2016;164:52-6.

3. Schneider CA, Rasband WS, Eliceiri KW. NIH Image to ImageJ: 25 years of image analysis. Nat Methods. 2012;9(7):671-5.

4. Schindelin J, Rueden CT, Hiner MC, Eliceiri KW. The ImageJ ecosystem: An open platform for biomedical image analysis. Mol Reprod Dev. 2015;82(7-8):518-29.

**Supplementary Tables**

**Table S4: Immunological phenotype**

|  | **Units** | **Patient** | | |
| --- | --- | --- | --- | --- |
| **Age** |  | **4y 2mo** | **6y 11mo** | **8y 11mo** |
| **T cells** | | | | |
| CD3^+^ | cells/µl (%)  *(normal range /µl)* | 3024 (66)  *(1400-3700)* | 1731 (66)  *(1200-2600)* | 2531 (65) *(1000-3300)* |
| CD4^+^ |  | 1741 (38) (*700-2200*) | 865 (33) (*650-1500*) | 1115 (27) *(650-220)* |
| CD8^+^ |  | 962 (21) *(490-1300)* | 656 (25) *(370-1100)* | 1080 (28) *(310-1200)* |
| CD4^+^CD45RA^+^CCR7^+^ | %  *(normal range %)* | 67  *(45-83)* | 48  *(34-79)* | 39  *(49-78)* |
| CD4^+^CD45RO^+/^RA^-^ |  | 29  (14-44) | 45  *(16-57)* | 58  (19-41) |
| CD8^+^CD45RA^+^CCR7^+^ |  | 60  *(33-87)* | 61  *(23-74)* | 44  (36-80) |
| **B cells** | | | | |
| CD19^+^ | cells/µl (%)  *(normal range /µl)* | 1283 (28) *(390-1400)* | 787 (30) *(270-860)* | 1154 (30) (*210-800)* |
| CD19^+^CD27^-^IgD^+^ | %  *(normal range %)* | **92**  *(70-88)* | **94**  *(*65-85) | **91.7**  (65-85) |
| CD19^+^CD27^+^ |  | **7.5**  *(9.8-23.3)* | **6.5**  *(12.4-30)* | **7.1**  *(12.4-30)* |
| CD19^+^CD20^+^CD27^+^IgM^+^ |  | **4.4**  *(5.2-13.4)* | **3**  *(6.8-18.7)* | **3.7**  *(6.8-18.7)* |
| CD19^+^CD20^+^CD27^+^IgM^-^ |  | **2.4**  *(3.1-14)* | **1.7**  *(4.2-16)* | **1.8**  *(4.2-16)* |
| IgA^+^ |  | **1.1**  *(1.2-3.7)* | **1.9**  *(1.5-6.1)* | **1.2**  *(1.5-6.1)* |
| IgG^+^ |  | **2.3**  *(2.7-13.9)* | **1.8**  *(3.9-14.7)* | **2.4**  *(3.9-14.7)* |
| **NK cells** | | | | |
| CD16^+^CD56^+^ | cells/µl (%)  *(normal range /µl)* | 238 (5.2) *(100-600)* | 102 (3.9) *(80-600)* | 199 (5)  *(80-600)* |
| **Immunoglobulins** | | | | |
| IgM | g/l  *(normal range g/l)* | 0.38  *(0.31-2.08)* | 0.17  *(0.31-2.08)* | 0.46  *(0.31-2.08)* |
| IgA |  | 1.35  *(0.34-3.05)* | 2.1  *(0.34-3.05)* | N/A |
| IgG |  | 8.8  *(5.7-14.7)* | **16.4**  *(5.7-14.7)* | **17.8**  *(5.7-14.7)* |
| **Lymphocyte proliferation** | | | | |
| IL-2 | % of control | 76 |  |  |
| PHA |  | 73 |  |  |
| CD3/CD8 beads |  | 79 |  |  |
| Tetanus |  | 2 |  |  |
| Candidin |  | 46 |  |  |
| Adenovirus |  | 26 |  |  |
| **Vaccination titers** | | | | |
| Tetanus | IU/ml | **0.06 (neg)** |  | **<0.01 (neg)** |
| Diphtheria |  | **<0.05 (neg)** |  | **<0.01 (neg)** |
| Streptococcus pneumoniae | mg/dl | **9.3 (low)** |  | 10.7 (pos) |
| Measles | AU/ml |  |  | 34.4 (pos) |
| Mumps |  |  |  | >300 (pos) |
| Rubella | IE/ml |  |  | 27.3 (pos) |

**Table S5: Sizes of *IKBKG* transcripts and NEMO proteins**

| **Transcripts** | **Amplicon size (bp)** | **Amino acids (aa)** | **Protein size (kDa)** |
| --- | --- | --- | --- |
| Wild type (exon 3-8) | 780 | 419 | 48 |
| Mutant (exon 3-8*) | 780 | 205 | 24 |
| Δ exon 4 (exon 3, 5-8) | 661 | 380 | 43 |
| Δ exon 5 (exon 3, 5-8) | 627 | 368 | 42 |
| Δ exon 4-5 (exon 3, 5-8) | 508 | 329 | 38 |
| Δ exon 4-6 (exon 3, 5-8) | 411 | 296 | 34 |

* Exon 5: c.613C>T (p.Gln205*)

**Table S6: NEMO function in peripheral blood cells obtained from the mother**

| **Stimulation** | | **Mother** | **Reference** |
| --- | --- | --- | --- |
| **IL-1β** | IL-6 (pg/ml) | 92 | >55 |
| **LPS** | IL-6 (pg/ml) | 3710 | >4384 |
| **TNFα** | IL-10 (pg/ml) | 36 | >18 |
| **PAM2CSK4** | IL-6 (pg/ml) | 439 | >182 |
| **PMA/Ion** | IL-6 (pg/ml) | 146 | >387 |
| **PMA/Ion** | IL-10 (pg/ml) | <5 | >9 |

**Table S7: Pixel densities obtained for cytokines/chemokines analyzed from Proteome Profiler Human Cytokine Array using the Protein Array Analyzer for Image J**

|  | **Control 1** | **Control 2** | **Mother** | **Patient** |
| --- | --- | --- | --- | --- |
| CCL5C5/C5aC | 5844,75 | 9101,25 | 7079,75 | 8402,25 |
| CD40 Ligand/TNFSF5 | 381,25 | 419,75 | 1528,75 | 3197,25 |
| C5/C5a | 1867,75 | 3004,75 | 343,75 | 6034,25 |
| CXCL1/GROα | 834,75 | 848,75 | 569,75 | 2566,25 |
| CXCL10/IP-10 | N/A | N/A | 6,25 | 1584,25 |
| CXCL11/I-TAC | N/A | N/A | N/A | 188,25 |
| CXCL12/SDF-1 | 1303,25 | 1209,75 | 937,75 | 1179,25 |
| ICAM-1/CD54 | 7141,25 | 8953,25 | 6934,25 | 8389,75 |
| IL-1ra/IL-1F3 | 491,75 | 223,75 | 423,25 | 2153,25 |
| IL-16 | 352,25 | 440,25 | 1,75 | 110,25 |
| IL-18/IL-1F4 | N/A | N/A | 52,75 | 2300,25 |
| MIF | 2804,75 | 1937,75 | 3269,25 | 2893,25 |
| Serpin E1/PAI-1 | 8689,75 | 9356,25 | 7653,75 | 8705,75 |

**Supplementary Figure Legends**

**Figure S1: *IKBKG* transcript variants and NEMO proteins**

*IKBKG* transcripts were amplified from exon 3-8. Exons, coding sequence (CDS) and primer binding sites are shown in (**A**). NEMO proteins were detected using two antibodies with binding sites at amino acids (aa)150-300, corresponding to exon 4-7, and aa278-396 (exon 7-10), respectively (**B**). *IKBKG* transcript variant 3 (NM_003639) was used as reference. RT-PCRs were performed on cDNA transcribed from T, B, NK lymphocytes and monocytes isolated from the patient (P), her mother (M) and 3 healthy controls (**C**). Shown are *IKBKG* transcripts observed by electrophoresis of RT-PCR products and a size marker. The full-lengths transcript cloned into the pcDNA6 vector served as positive control (pos), a PCR product without template was loaded as negative control (neg) to exclude contamination. *IKBKG* transcripts wild type (wt), containing mutation c.613C>T (mut), ∆exon4 (∆4), ∆exon5 (∆5), ∆exon4-5 (∆4-5), ∆exon4-6 (∆4-6) were cloned into the pcDNA6 vector. Corresponding proteins were synthesized *in vitro* and used to test binding of abNEMO^150-300^ und abNEMO^278-396^ by western blotting (**D, E**). Besides the wt variant, the mut variant could be detected by abNEMO^150-300^ (**D**), whereas abNEMO^278-396^ bound to ∆5, and ∆4-6, but not mut (**E**). No protein is translated from ∆4 and ∆4-5 variants due to disruption of the reading frame. GAPDH was used as loading control.

**Figure S2: NEMO^dim^ and NEMO^bright^ populations can be detected using flow cytometry**

NEMO proteins were analyzed in peripheral blood cell subsets using flow cytometry. T (CD3^+^), B (CD19^+^), NK (CD3^-^CD56^+^) lymphocytes and monocytes (CD14^+^) were identified by surface marker staining as shown by the gating strategy in (**A**). (**B**) In addition to the analysis shown in Figure 3D, intracellular NEMO protein expression was studied in patient monocytes, granulocytes, CD4^+^ and CD8^+^ T cells, and NK cells at two different time points (age of 4y and 5y) using abNEMO^aa278-396^ in the diagnostic immunology laboratory of Labor Berlin, Charité Berlin, Germany. Fluorescence intensities are shown as histograms. The isotype is color-coded in grey, patient cells in red, and results from a healthy control are shown in blue. (**C**) The transcript variants WT, ∆ex5, and WT+∆ex5 cloned into the pcDNA6 vector were transfected into HCT116 colon carcinoma cells. NEMO protein expression was investigated in untransfected cells (endogenous expression), as well as in transfected cells using flow cytometry. Shown are fluorescent intensities as histograms of isotype (grey), endogenous NEMO (light blue), ∆ex5 transfected (orange), WT transfected (dark blue) and WT+∆ex5 transfected (pink) cells following intracellular staining with abNEMO^aa150-300^ and abNEMO^aa278-396^, respectively. (**D**) Mean fluorescent intensities were normalized on isotype and endogenous NEMO expression and are shown as diagrams for abNEMO^aa150-300^ (left panel) and abNEMO^aa278-396^ (right panel). Shown are means and standard deviations of three experiments. (**E**) NEMO^bright^ (NEMO^+^) and NEMO^dim^ (NEMO^-^) PBMCs obtained from the patient were sorted (left panel) after intracellular staining using abNEMO^278-396^. RNA was isolated and transcribed into cDNA. Non-fixed patient PBMCs served as control. Exon 3-7 was amplified by PCR (center) and products visualized by electrophoresis were identified by Sanger sequencing (right). Shown is one representative result of 3 experiments.

**Figure S3: Elevated cytokines/chemokine levels can be found in patient serum**

The Proteome Profiler Human Cytokine Array Kit Panel A was used to test the expression of 36 cytokines in serum samples obtained from the patient, her mother and control 1 and 2. The membranes were developed as described for western blotting. Each cytokine, or control protein, respectively, is represented by two spots. Shown are the membranes and cytokines/chemokines with increased expression in patient serum compared to the other samples marked by color coded squares. Corresponding cytokines are listed on the right-hand side.

**Figure S4: The DNA methylation status of regions adjacent to *IKBKG* differs between mother and patient**

DNA methylation of patient, mother and father observed in the neighboring regions of *IKBKG* (chrX:154,483,311-154,570,277 in hg38) by Oxford Nanopore Sequencing (ONT) is displayed in the Integrative Genomics Viewer (IGV). The stacked bars indicate the number of reads indicating presence (red) and absence (blue) of DNA methylation. Differences in the DNA methylation between mother and patient are depicted within the black boxes.

**Figure S5: Reversion mosaicism was excluded in peripheral blood cell subsets**

Genomic DNA was isolated from separated T, B, NK lymphocytes, monocytes, and granulocytes obtained from the patient and her mother. To exclude reversion mosaicism in cellular subsets, gDNA was sequenced by Sanger sequencing. The monoallelic point mutation could be observed in T, B, NK lymphocytes, as well as monocytes and granulocytes isolated from both patient and mother.
